# *Ardissonea crystallina* has a type of sexual reproduction that is unusual for centric diatoms

**DOI:** 10.1038/s41598-017-15301-z

**Published:** 2017-11-07

**Authors:** Nickolai A. Davidovich, Olga I. Davidovich, Yulia A. Podunay, Romain Gastineau, Irena Kaczmarska, Aloisie Poulíčková, Andrzej Witkowski

**Affiliations:** 1 T. I. Vyasemsky Karadag scientific station – Nature Reserve, village Kurortnoe, Feodosiya, 298188 Russia; 2 MMS EA 2160, Faculté des Sciences et des Techniques, Université du Maine, Avenue Olivier Messiaen, 72085 Le Mans, Cedex 9 France; 3 Department of Biology, Mount Allison University, Sackville, New Brunswick E4L 1G7 Canada; 4 Department of Botany, Faculty of Science, Palacký University in Olomouc, Šlechtitelů 27, CZ-78371 Olomouc, Czech Republic; 5 Natural Sciences Research and Educational Center and Palaeoceanology Unit, Faculty of Geosciences, University of Szczecin, Mickiewicza 16a, Szczecin, 70-383 Poland

## Abstract

Molecular phylogenetic analyses place *Ardissonea crystallina* (C. Agardh) Grunow and all Toxariids among the bi- and multipolar centric diatoms, almost always recovered as a derived lineage sister to *Lampriscus*. In all centrics where sexual reproduction has been documented, oogamy, with larger immobile eggs and smaller flagellated sperm has been observed. We were able to initiate both homothallic and heterothallic reproduction in *A. crystallina*. The heterothallic reproduction turned out to be non-oogamous; gametes were more or less equal in size but no flagellated cells were detected. At the same time, two mating types (“male” and “female”) were recognized by the distinct morphology and behaviour of the gametes. While no flagella were observed, periodically thin cytoplasmic projections arose on the surface of the “male” gametes. These projections similar to those found in some pennate diatoms facilitated contact with the “female” cells. In each gametangial cell, regardless of the mating type, only one gamete was formed. Thus, the Toxariids may represent a unique evolutionary group, at least in respect to their reproductive biology. The hypothesis discussed is that non-oogamous mode of reproduction could have evolved in *Ardissonea* (and possibly in other Toxariids) independently of the pennate lineage of diatoms.

## Introduction

Historically, the diatoms have been divided into two major groups, centrics and pennates^1^, based primarily on the pattern of symmetry of their frustules, mode of sexual reproduction and plastid number and structure^2^. Centric diatoms have radially-symmetrical valves, typically exhibit multiple plastids and, in taxa where sexual reproduction has been observed, reproduce oogamously, with larger immobile ova and smaller, flagellated sperm cells. In contrast, pennate diatoms were classified by their generally bilaterally-symmetrical valves, fewer plate-like plastids and non-oogamous sexual reproduction, involving non-flagellated, but still occasionally motile, gametes. This system had been accepted well into the last century, though with an additional division of pennates into “araphid” and “raphid”^3,4^.

The introduction of molecular data to phylogenetic analyses led to revisions of higher level diatom systematics. Molecular data^5–9^ have questioned the centric–pennate bipartition established by earlier, morphological-based research. Based on rDNA sequence-based phylogenies and supported by reproductive and cytological characters, Medlin and Kaczmarska^5^ proposed a classification system in which the diatoms (division Bacillariophyta) were separated into two subdivisions: Coscinodiscophytina (comprised of most of the radially-symmetrical centric diatoms) and Bacillariophytina (comprised of the diatoms which exhibit polarity in the shape of their valves). The subdivision Bacillariophytina was further divided into two classes: the Mediophyceae and Bacillariophyceae. The class Mediophyceae includes the bi/multi polar centric diatoms and the radial Thalassiosirales, whereas all pennate diatoms, both raphid and araphid, belong in the class Bacillariophyceae.

In this context, a very interesting situation involves a clade, which contains genera of *Ardissonea* De Notaris, *Climacosphenia* Ehrenberg, and *Toxarium* De Notaris. Taxa belonging to this clade display some morphological and cytological features that are usually attributed to centrics: they possess many discoid chloroplasts, do not have obvious apical pore fields, and median sterna. At the same time, they have extremely-elongate valves, and an attached habit, like many pennate diatoms. Motility, which is observed in pennate taxa but not in centric, has, however, been documented in *Ardissonea* and *Toxarium*, but in the vertical position of the cell in relation to a substrate^10,11^. Whereas the general morphology, ecology and behavior of these genera is equivocal about the systematics and classification of these genera, the DNA data strongly support their derived position among bipolar and multipolar centrics^8,9,11–14^. A recently published six-gene phylogeny shows Toxarriids immediately preceding the pennates^15^.

Sexual reproduction features may provide significant insight into understanding the evolutionary relationship between *Ardissonea* and allied taxa *Toxarium* and *Climacosphenia* to other polar centrics and pennates. However, it has not been investigated thus far in any member of this clade. This gap in our knowledge has already been pointed out by some authors^8,16^. We were able to initiate sexual reproduction in *Ardissonea crystallina* (C. Agardh) Grunow maintained in culture. Here we document the course of sexual reproduction in this exceedingly elongated, non-pennate centric polar diatom and discuss the evolutionary context of this process.

## Results

### Life form and life cycle

**Figure 1:**
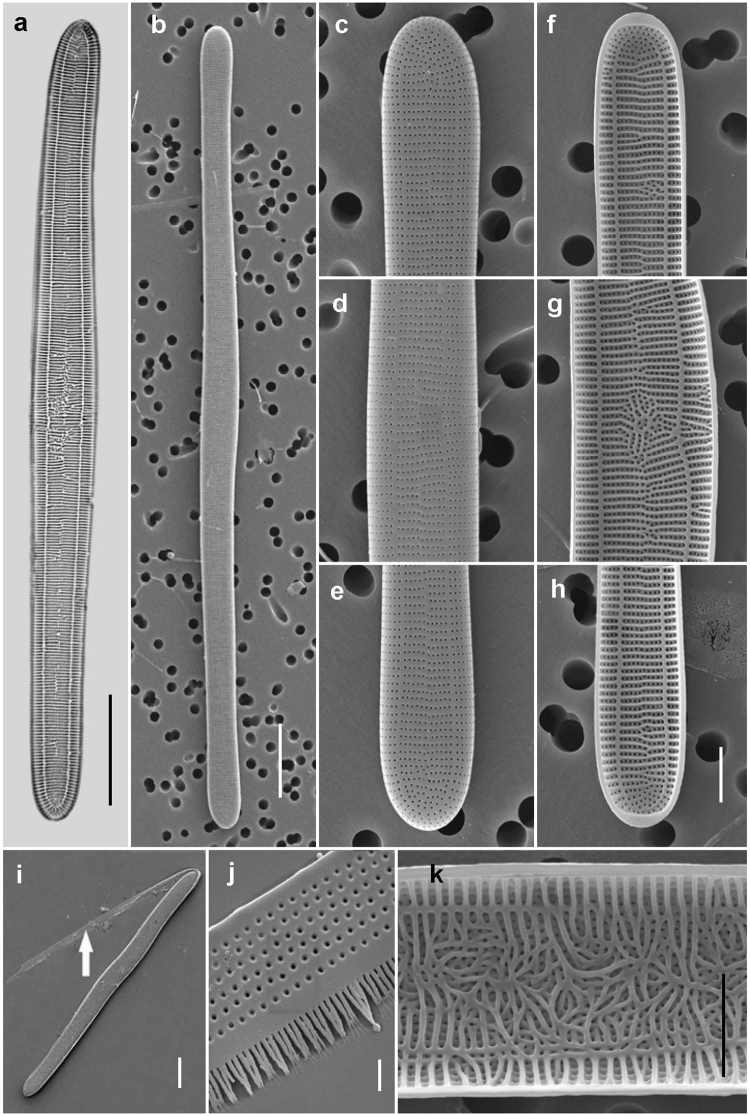
Cleaned valves of *Ardissonea crystallina* (C. Agardh) Grunow thecae observed using (**a**) light microscope and (**b**–**h**) scanning electron microscope. (**a**) Internal and (**b**) external views of the whole valve. (**c**–**e**) External and (**f**–**h**) internal views of the apical and middle parts of the valve. (**i**) Valve with dehisced girdle band (arrow). (**j**) A part of the band indicated by the arrow in (**i**) at higher magnification. (**k**) The inner central part of the valve may have very intricate tracery (compare with g). Scale bars (**a,b,i**) = 20, (**c–h,k**) = 5, (**j**) = 1 µm.

**Figure 2:**
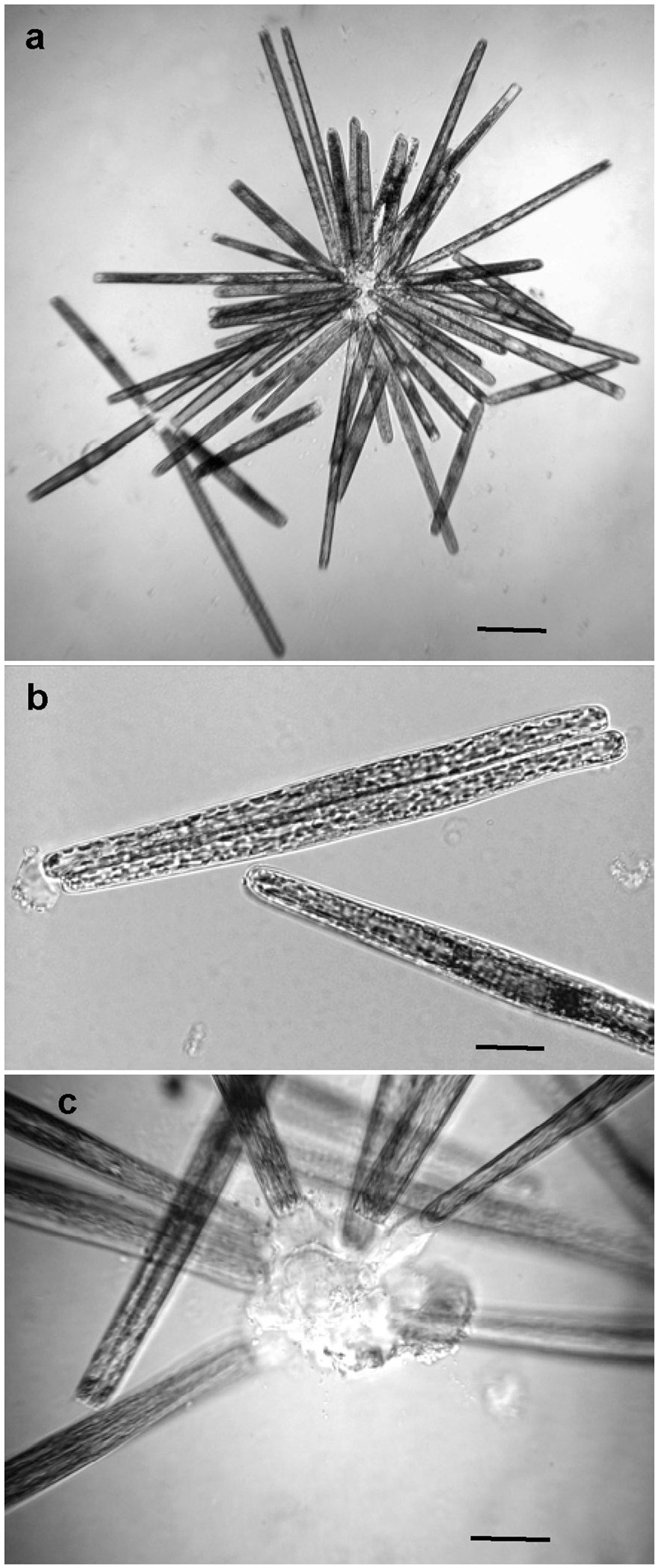
*Ardissonea crystallina* (C. Agardh) Grunow growth form by light microscopy, differential interference contrast. (**a**) A colony constituted by cells of two different size classes; differently-sized cells represent two different clones. (**b**) Multiple discoid chloroplasts dispersed throughout the cell cytoplasm. (**c**) Cells in the colony are attached by one end to the common mucilage pad secreted through the cell apex. Scale bars (**a**) = 100; (**b,c**) = 20 µm.

The diatom being investigated was identified as *Ardissonea crystallina* (C. Agardh) Grunow based on the morphology and structure of frustules (Fig. 1), the type of chloroplasts, the growth habit (Fig. 2), and molecular phylogeny (see below). In nature, the cells are benthic, either epiphytes or on inorganic substrates, growing separately or more often forming dense colonies (Fig. 2a). Each cell in a colony secreted abundant mucilage from the cell pole to form a common mucilage pad (Fig. 2c). The cells, however, had no specialized apical pore fields on the valve suggesting a source of mucilage secretion (see Fig. 1c,e,f,h). Rimoportulae and a distinct sternum were also absent. The cell apical length in the source population, inhabiting Bukhta Kazachya (Sevastopol, Crimea) and sampled on July 2011, ranged from 117 to 498 µm; most abundant (one fifth of the total count; total *N* = 441) were cells *ca*. 340 µm long. The cell-size distribution was bell-shaped (Anderson-Darling normality test produced a confidence level of 77.76%); the distribution was not skewed and there were no peaks corresponding to large cells suggesting recent auxosporulation events (Supplementary Fig. S1, Supplementary Material). The size range of the cells maintained in culture varied by an order of magnitude, from 678 µm (the largest initial cell) to 76 µm (the smallest vegetative cell; Supplementary Tables S1 and S2, Supplementary Material). When growing in culture, cell apical length decreased uniformly in all the clones (Supplementary Fig. S2, Supplementary Material), at an average rate of about 7.1–10.5 µm/month (mean ± SE = 8.6 ± 0.3, *N* = 11). The cardinal point corresponding to the upper limit of the sexually inducible size range (*sensu* Geitler^17,18^) was around 304 µm, i.e. 45% of the maximal species-specific cell length (Supplementary Table S3, Supplementary Material). In theory, the average time from the beginning of the life history to entry into the reproductive stage was thus (678-304)/8.6 = 44 months, i.e. 3.7 years. In reality, in the early part of the life cycle, particular cells in clones decreased over several months by 20–30%. With time, because not all the cells underwent such rapid size reduction, several size classes could be found in some clones. The pre-reproductive period became shorter for these smaller cells. There was a weak positive relationship between initial cell length and parental cell length (Supplementary Fig. S3, Supplementary Material).

### Breeding system

Results of sexual reproduction were first detected in clone 1.0721-Q one month after isolation from the natural sample. The entire process of intraclonal reproduction was not observed at that time; only the resulting initial cells were found. One month later, mating of suitable (designated as “sexually compatible”) clones revealed a heterothallic mode of reproduction. During the next six years, new clones isolated from the same locality were sexually compatible with the existing clones if they had an appropriate mating type and their cell length was within the sexually inducible size range. All the clones derived from the natural population were involved in heterothallic mating (Supplementary Table S4, Supplementary Material). Descendant clones resulting from intra- and interclonal reproduction were also able to reproduce heterothallically. In contrast to the heterothallic mode, homothallic reproduction was infrequent and much less abundant.

Only 12 of 30 “wild” clones (isolated from the natural population) underwent intraclonal reproduction, and surprisingly, almost all these clones were of the same mating type, designated as “mt2”. In this mating type, gametes did not escape from the gametangial cells and filled them almost entirely (stationary gametes). In contrast, ball shaped gametes produced by “mt1” clones released into the surrounded space were motile (see below). Recognition of two sexes, male and female, corresponded to “mt1” and “mt2” mating types is thus well justified morphologically and behaviourally. Excepting two cases when auxospores were found in the mixtures of two “mt1” clones, the last did not reproduce intraclonally. Gametogenesis in pairs of clones usually started on the fifth or sixth day after two sexually compatible clones were mixed. Intraclonal gametogenesis occurred generally on the sixth or seventh day after the clone was reinoculated into fresh growth medium.

### Sexual reproduction

After reinoculation, the cells settled on the bottom of Petri dishes in an apparent random fashion and formed mitotically dividing colonies. In mixtures, cells of two clones (distinguishable by cell sizes) may form colonies comprised of longer and shorter cells (Fig. 2a). This may occur in two ways: (1) either cells of one clone could randomly settle down in close proximity to cells of the other clone, or (2) this could have been the result of purposeful movement. We cannot exclude the possibility that male and female cells became more physically closely associated with each other than would be expected by chance alone. However, no obvious pairing of parental cells similar to that typical of raphid pennates was ever seen in clone mixtures despite the ability of cells to move slowly.

**Figure 3:**
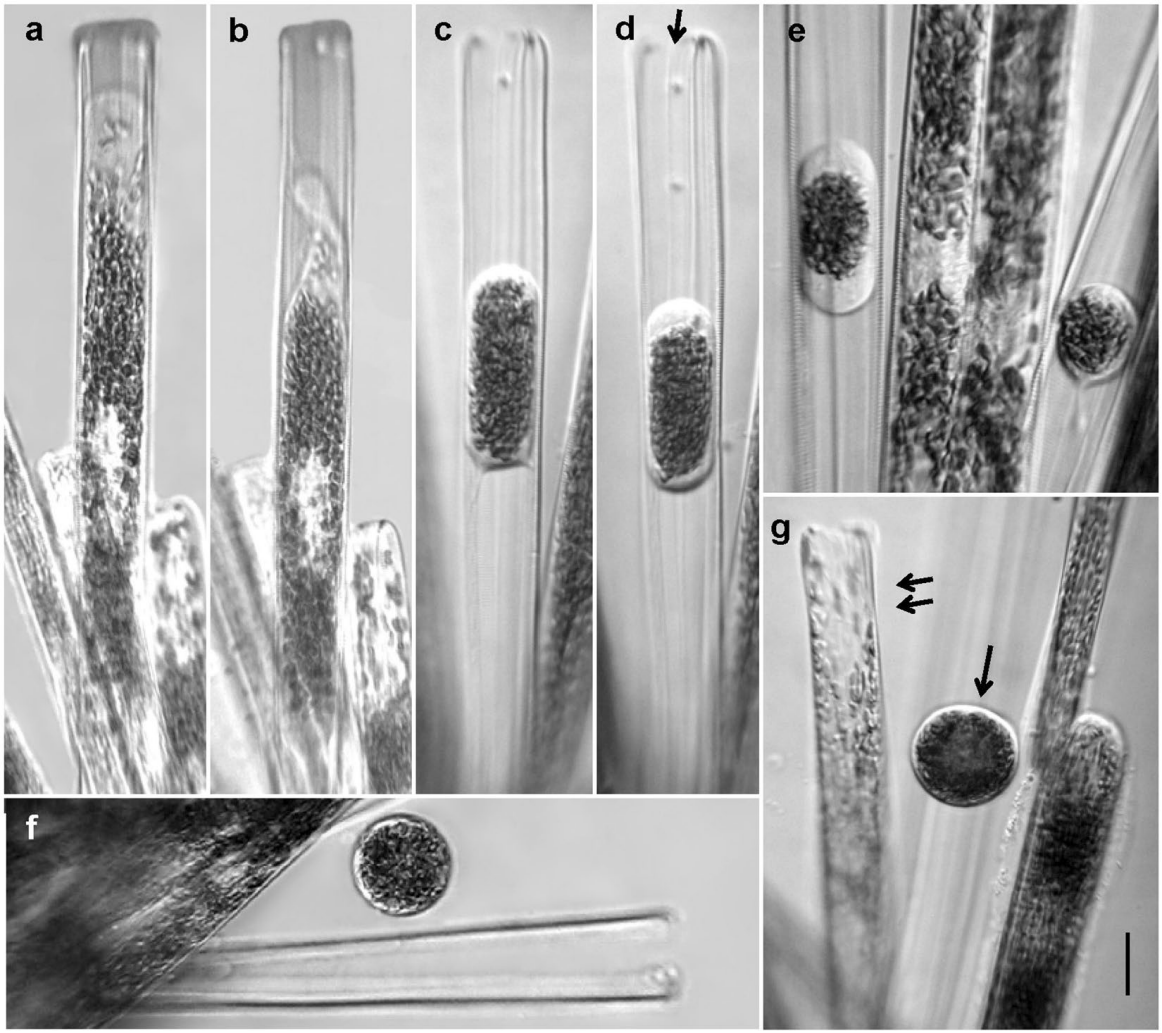
Male gametogenesis in *Ardissonea crystallina* (C. Agardh) Grunow. (**a**–**d**) The contents of the male gametangium shrink from both ends of the cell to form an ellipsoidal gamete positioned in the centre of the gametangial frustule. The gamete transapical dimension increases and as a result the gametangial thecae becoming slightly opened (arrow). (**e**) Chloroplasts are densely aggregated in the remaining cytoplasm. (**f**) Finally, the male gamete becomes spherical, in contrast to (**g**) the female gamete which does not escape from the gametangium frustule and fills it (arrow: male gamete, double arrow: female gamete). Scale bars (**a–g**) = 20 µm.

After several days of growth, sexualized cells in the colonies proceeded to the meiotic cycle and produced gametes. Male and female gametogenesis differed cytologically, but they were similar in that only one gamete was produced per gametangium. After the first meiotic division, the contents of the male gametangia contracted and took a position in the cell center (Fig. 3). In the contracted male gamete, chloroplasts were extremely densely aggregated. In rare cases, a residual body of cytoplasm was found within the thecae of the male gametangium. Spherical male gametes were released from, but remained close to, empty gametangial frustules. They had no flagella and did not move in the manner typical of the spermspermatozoids of centric diatoms.

**Figure 4:**
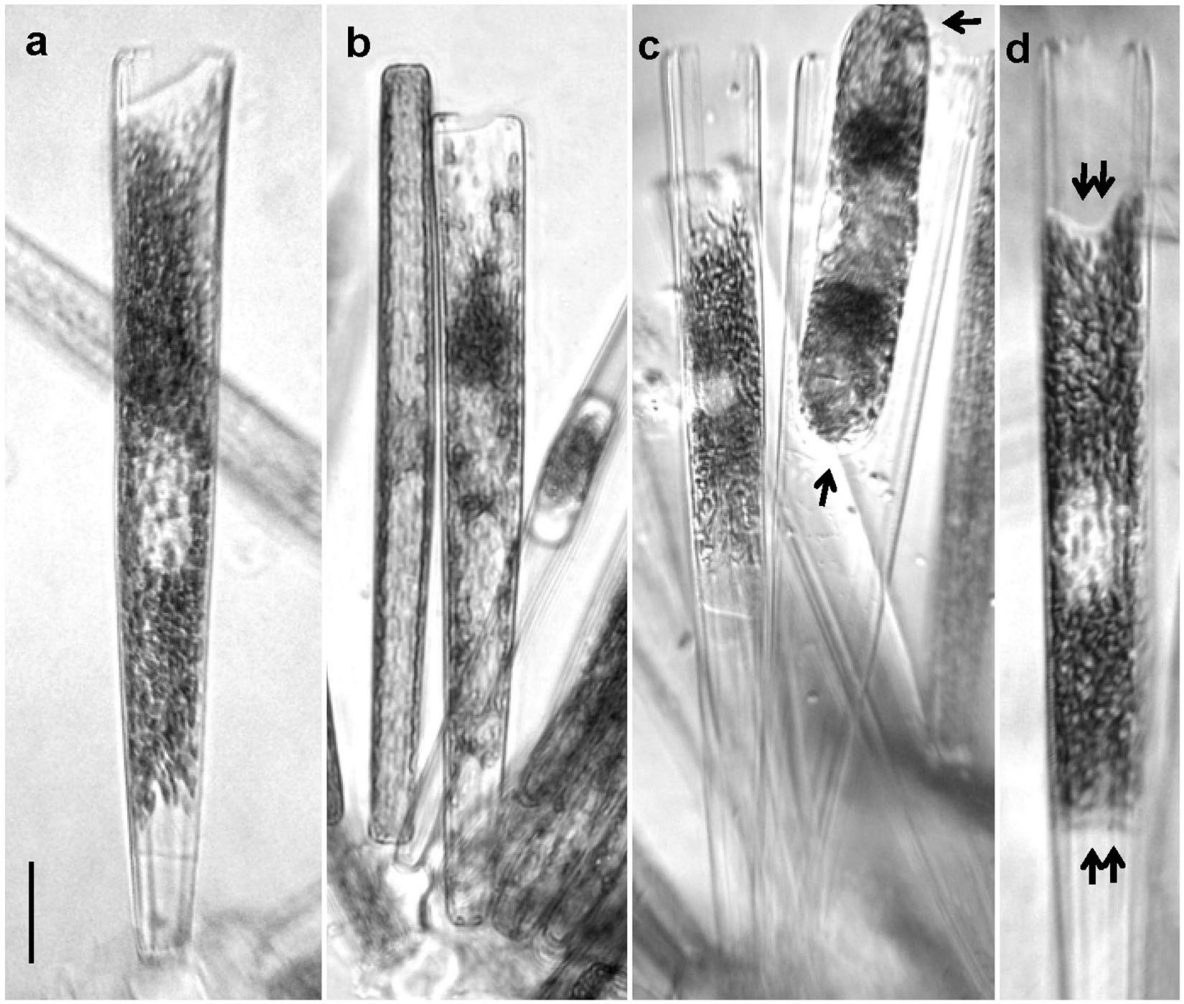
Female gametogenesis in *Ardissonea crystallina* (C. Agardh) Grunow. (**a**,**b**) The female gamete fills the entire gametangium frustule, which becomes slightly opened at the distal end. (**c**,**d**) If not fertilized, the female gamete retreats from the ends towards the cell centre. Note the difference between the ends in the zygote (arrows) and in the female gamete (double arrows). Scale bars (**a**–**d**) 20 µm.

The gametes produced by female gametangia were not easily distinguishable from dividing vegetative cells, the former being recognizable only by the cells being slightly opened at one apex (Fig. 4). The protoplast of female gametes stuck to the inner surface of the frustule, similar to a “wetting liquid” in a capillary tube. In contrast to females, the protoplast of male gametes at early stage of their formation looked as a “non-wetting liquid” (see Fig. 3).

**Figure 5:**
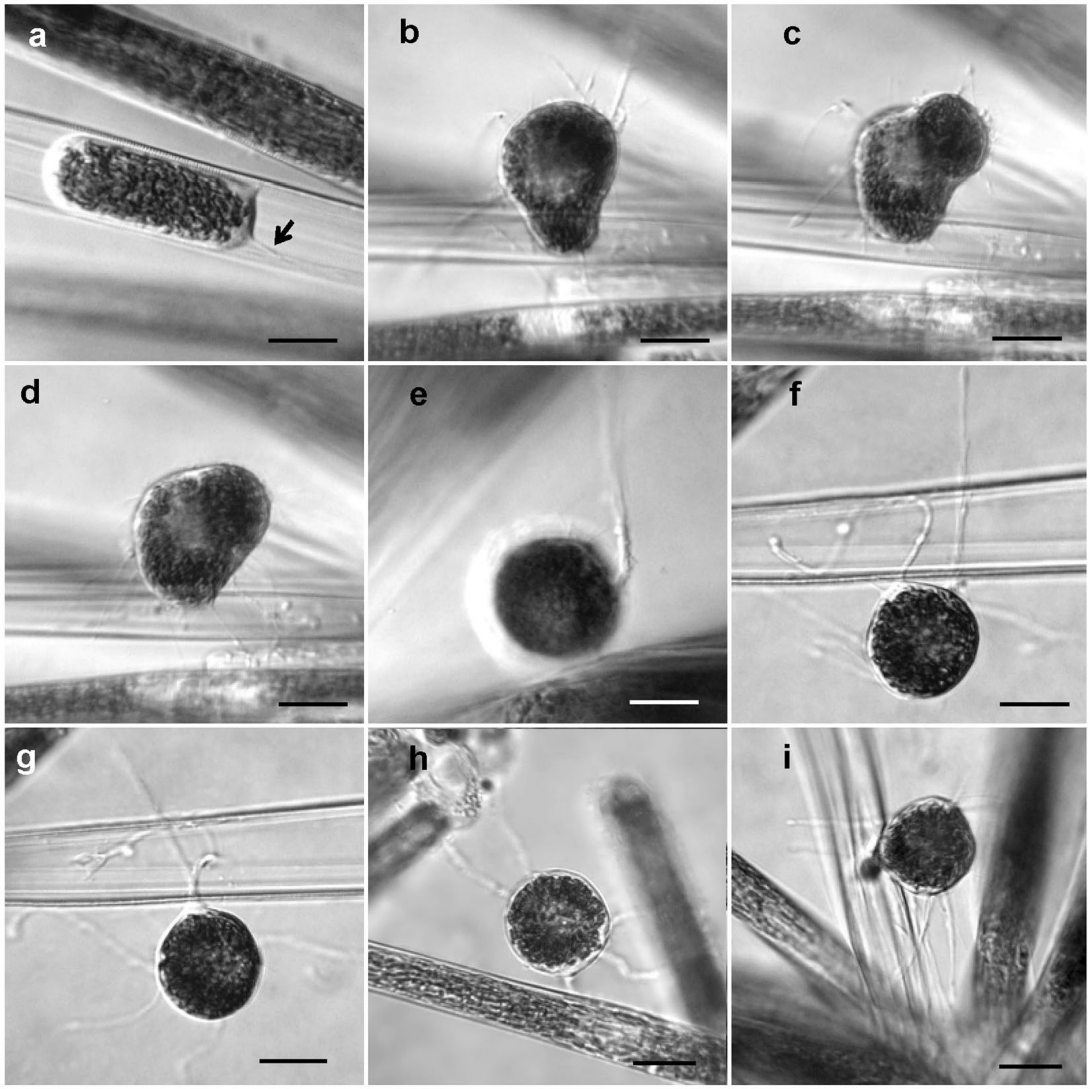
Formation of cytoplasmic projections in male gametes of *Ardissonea crystallina* (C. Agardh) Grunow. (**a**) Cytoplasmic projection (arrow) can be seen at the early stages of gamete formation. (**b**–**d**) The number and position of cytoplasmic projections may change relatively quickly. (**e**) Sometimes several projections stick together. (**f**,**g**) Cytoplasmic projections are very flexible. (**h**, **i**) Cytoplasmic projections connect to nearby cells. Scale bars (**a–e**) = 20 µm.

**Figure 6:**
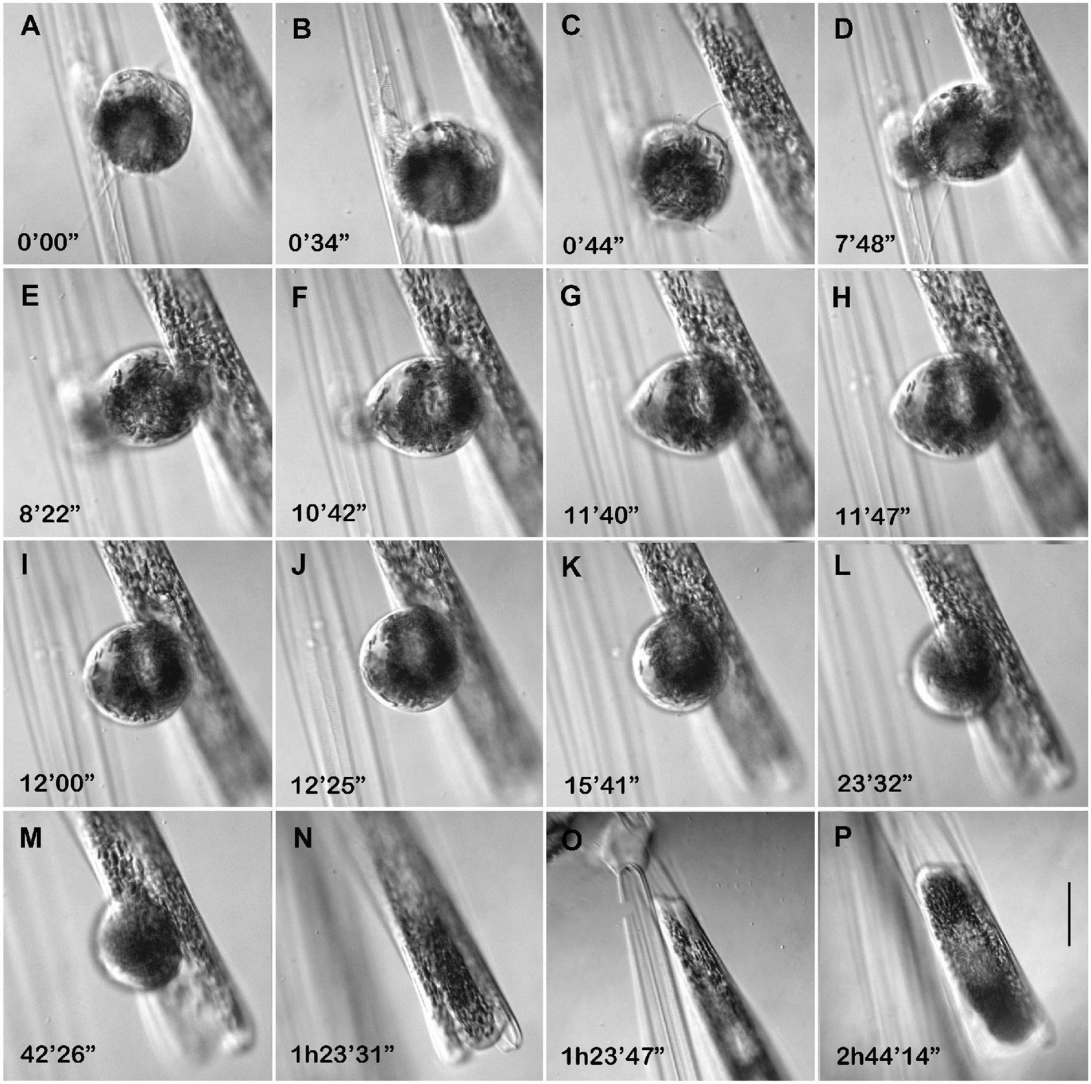
The process of syngamy in *Ardissonea crystallina* (C. Agardh) Grunow. (**a**–**c**) The male gamete released from the gametangium produces pseudopodia. (**d**–**j**) The male gamete moves from male to female gametangium. (**k**–**m**) The male gamete migrates to the distal end of the female gametangium up to the place where the gap between the thecae is sufficient for penetration. (**n**) Male and female gametes have fused. (**o**) The basal end of the same cell as in the previous image. (**p**) The zygote. Scale bars (**a–p**) = 20 µm.

Male gametes made contact with parental thecae and other neighbouring cells by thin cytoplasmic projections, whose form and behaviour resembled pseudopodia (Fig. 5). With the help of these cytoplasmic projections the male gametes could make contact with female gametangia. The male gamete reached the surface of the female gametangium, attached to it and then moved slowly to the slightly open distal apical end (Fig. 6). The locomotion of the male gamete on the surface of the female gametangial frustule resembled amoeboid movement. The male and female gametes merged upon reaching by the male gamete the point where the gap between the thecae of the female frustule was sufficient for penetration. The fusion took a minute or less.

**Figure 7:**
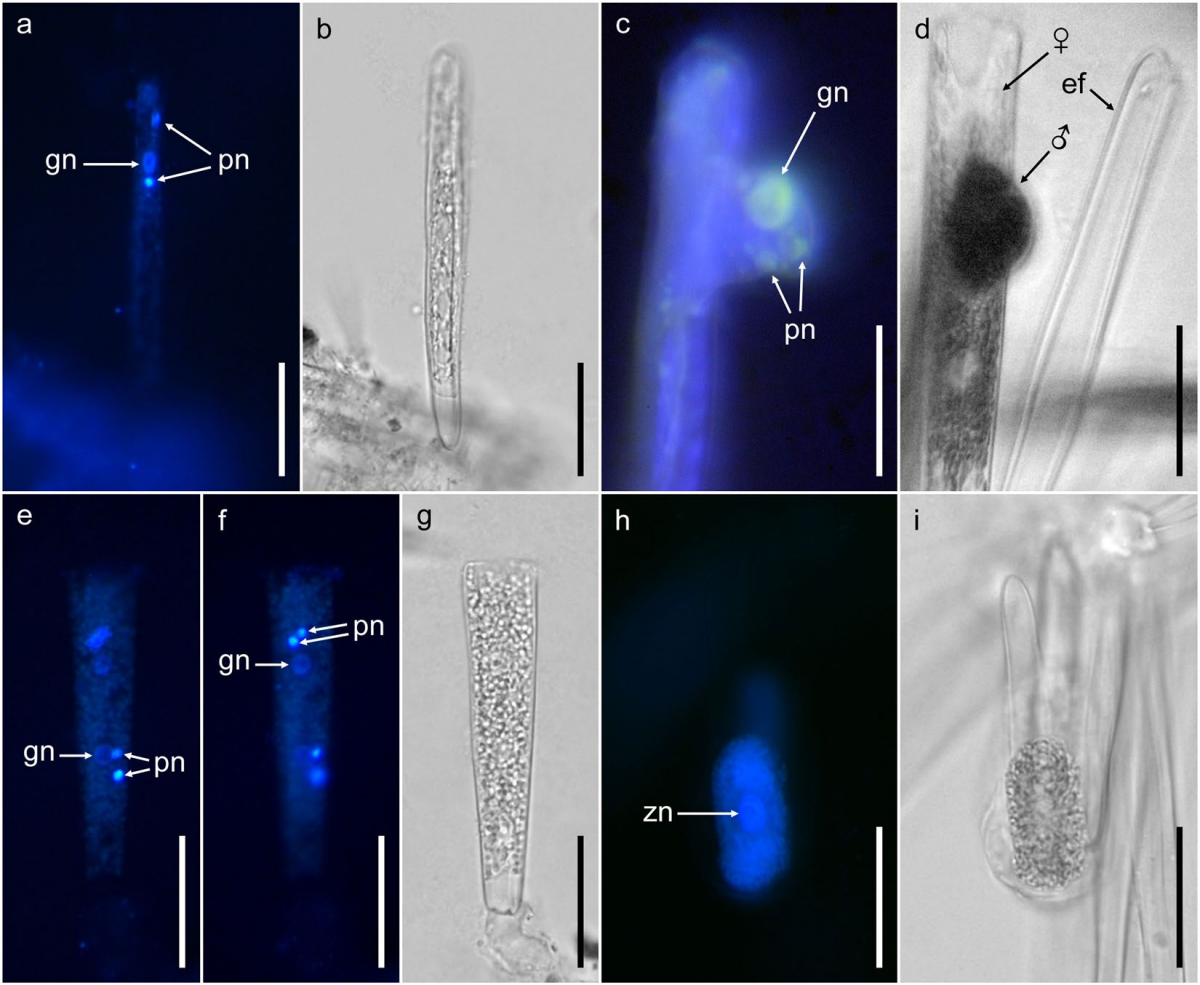
Nuclei in gametes and young auxospores of *Ardissonea crystallina* (C. Agardh) Grunow, epifluorescence (DAPI), brightfield and differential interference (DIC) microscopy. (**a**) Female gametangium with receptive gamete, note one gametic (gn) and two pyknotic (pn) nuclei (DAPI). (**b**) Brightfield image of the cell shown in (**a**) showing slightly opened frustule with only the top valve in focus. (**c**) Gamete fusion in progress (DAPI). Note one gametic (gn) and two pyknotic (pn) nuclei. (**d**) DIC image of a different cell undergoing gamete fusion, in an analogous position to (**c**). The male gamete penetrates the gap between thecae of the female frustule to contact the female gamete. In contrast to the male, the female gamete never leaves its gametangium before fertilization. In close proximity to the female gametangia, one can see empty frustules of the presumed male gametangium (ef). (**e**) Gamete fusion completed (DAPI). (**f**) The same cell shown in (**e**) with a different focal plane (DAPI). Together with (**e**), nuclei can be seen clustered in two groups of three, each with one functional, larger gametic (gn) and two smaller pyknotic (pn) nuclei. (**g**) Brightfield image of the same cell in (**e**,**f**), showing the open frustule of the six-nucleated cell. (**h**) Small auxospore with large diploid (zn) nucleus (DAPI). (**i**) Brightfield image of the same cell shown in (**i**). Scale bars (**a–i**) = 50 µm.

DAPI staining demonstrated nuclear behaviour during gametogenesis and fertilisation. Both gametes underwent two acytokinetic nuclear divisions resulting in one gametic and two pyknotic nuclei each (Fig. 7a–d). Following syngamy (Fig. 7c–g), the zygote contracted and only one nucleus could be seen there and in expanding auxospores (Fig. 7h,i).

**Figure 8:**
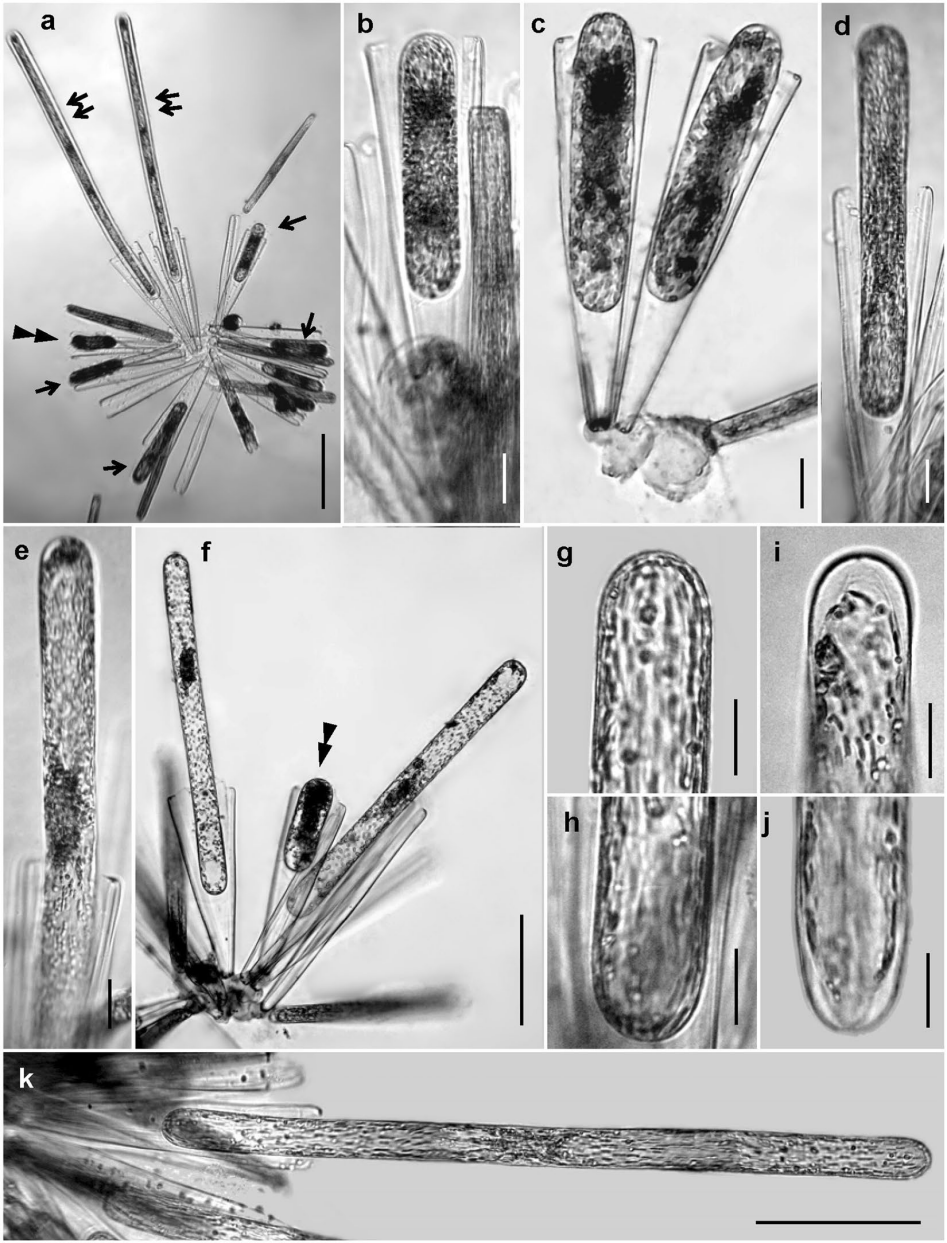
Auxospores in *Ardissonea crystallina* (C. Agardh) Grunow. (**a**) Auxospores at different stages of growth (arrows); young (double arrowhead) and fully expanded auxospores with initial cells inside (double arrows). (**b**) Very young auxospore within the bounds of the gametangial frustule; note the position of the gametangial distal end. (**c**–**f**) Young auxospore (double arrowhead) and larger auxospores. (**g**,**h**) In the growing, auxospores the cells ends are filled with cytoplasm. (**i**,**j**) In the mature auxospores, before initial cell formation, the cytoplasm shrinks slightly from both ends of the cell. (**k**) Fully expanded auxospore (montage of three images). (**c**,**f**) Cells are stained by haematoxylin. Scale bars (**a,f,k**) = 100; (**b–e, g–j**) = 20 µm.

**Figure 9:**
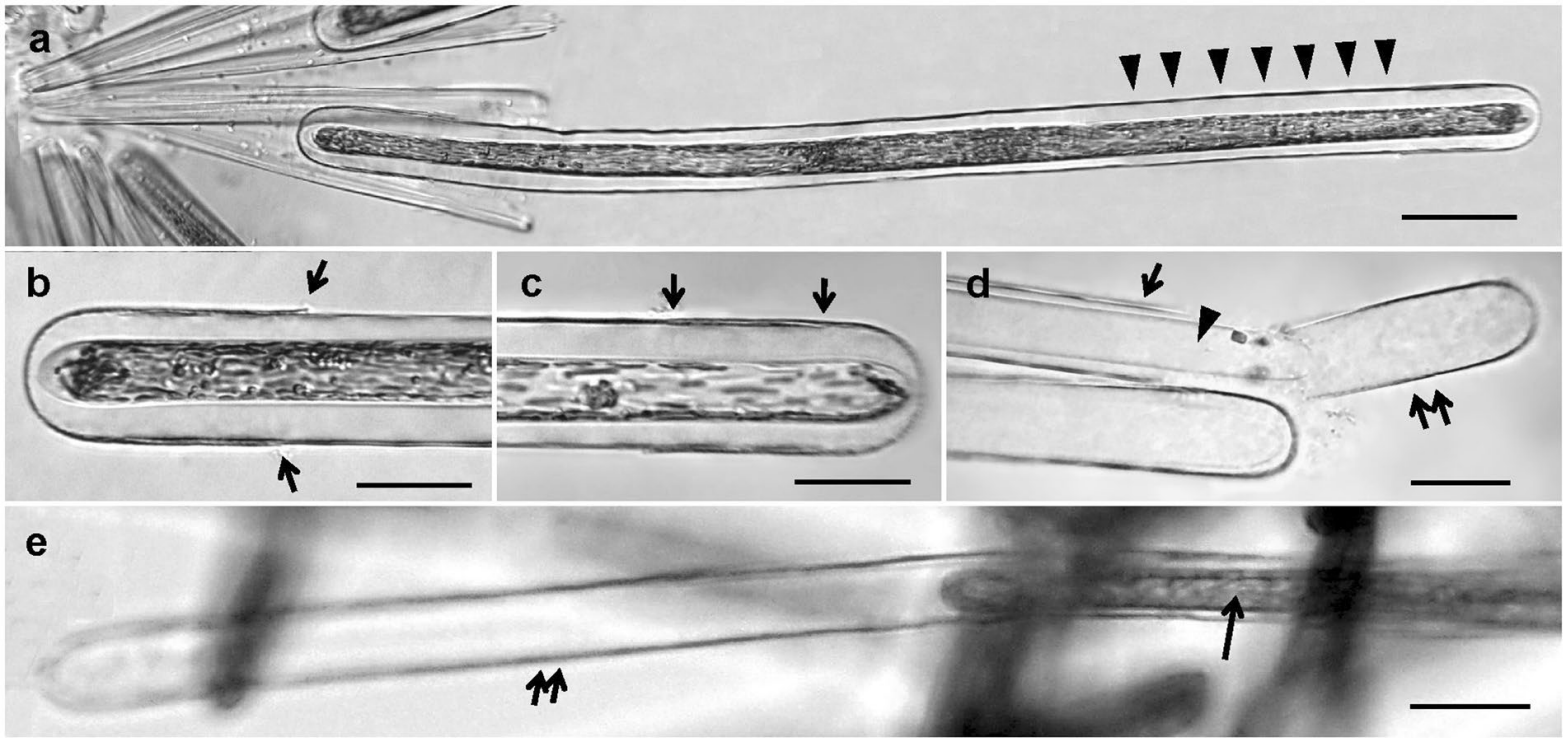
Initial cells in *Ardissonea crystallina* (C. Agardh) Grunow. (**a**) Initial cell inside the auxospore envelope (montage of four images), note the gap between the initial cell and the auxospore envelope, arrowheads indicate transverse elements. (**b**,**c**) Both ends of the auxospore are covered with the additional presumably siliceous elements. (**b**) The arrows indicate the edge of this additional siliceous element. (**c**) The arrows indicate overlapping distance of the initial cell and the additional, presumably siliceous element. (**d**) Dead cells; transverse elements (arrow), initial cell (arrowhead), and the additional siliceous element (double arrow) are seen in the broken cell. (**e**) Initial cell (arrow) escaping from the auxospore wall (double arrow) by active gliding. Scale bars (**a,e**) = 50; (**b–d**) = 20 µm.

Zygotes in *Ardissonea* were elongated and lay within the slightly opened frustule of the female gametangium (Figs 7i and 8). The general morphology of contracting zygotes was very similar to that of young auxospores. The smallest progeny cell with a diploid nucleus found in DAPI stained material was 42 um long, but there might have been even smaller cells. The auxospore expanded in a bipolar fashion along the longitudinal axis of the gametangial frustule, shifting during expansion in the direction of the distal end; as a result, the auxospore protruded from the parental frustule (Fig. 9). In young auxospores, chloroplasts grouped into two clusters on either side of the centre of the cell (Fig. 8b). During further auxospore growth, chloroplasts were evenly distributed across the cytoplasm. In the mature auxospores, before initial cell formation, the contents of the auxospore contracted and detached from the rigid envelope, starting from both ends of the cell.

Elongated auxospores were covered with an envelope that appeared to contain transverse perizonium-like structures. The envelope was rigid enough to provide bidirectional expansion of the very long (up to 680 *µ*m) auxospore. Transverse elements detectable in light microscopy were positioned approximately every 15–20 µm. Fine structural detail of the auxospore envelope is beyond the scope of this investigation and will be discussed elsewhere. After maturing, the initial cell escaped from the perizonium through its open end by active gliding, casting off the cap in the process. Similar mode of escaping from the perizonium is known in members of raphid pennate diatoms, e.g. *Haslea ostrearia* (Bory) Simonsen^19^, *H. karadagensis* ^20^, and *Nitzschia longissima* (Brébisson ex Kützing) Grunow^21^.

As mentioned above, intraclonal auxosporulation was also observed in some clonal cultures. Unfortunately, we were unable to determine whether these auxospores arose through allogamy or autogamy, as earliest stage directly observed in those cases was the auxospore. It was however possible to find empty frustules occurring presumably as the result of male gametogenesis close to the growing auxospores, themselves surrounded by valves of the maternal frustule. Altogether, this suggests an allogamous, thus homothallic, self-fertile reproduction.

### Species identity and phylogeny

**Figure 10:**
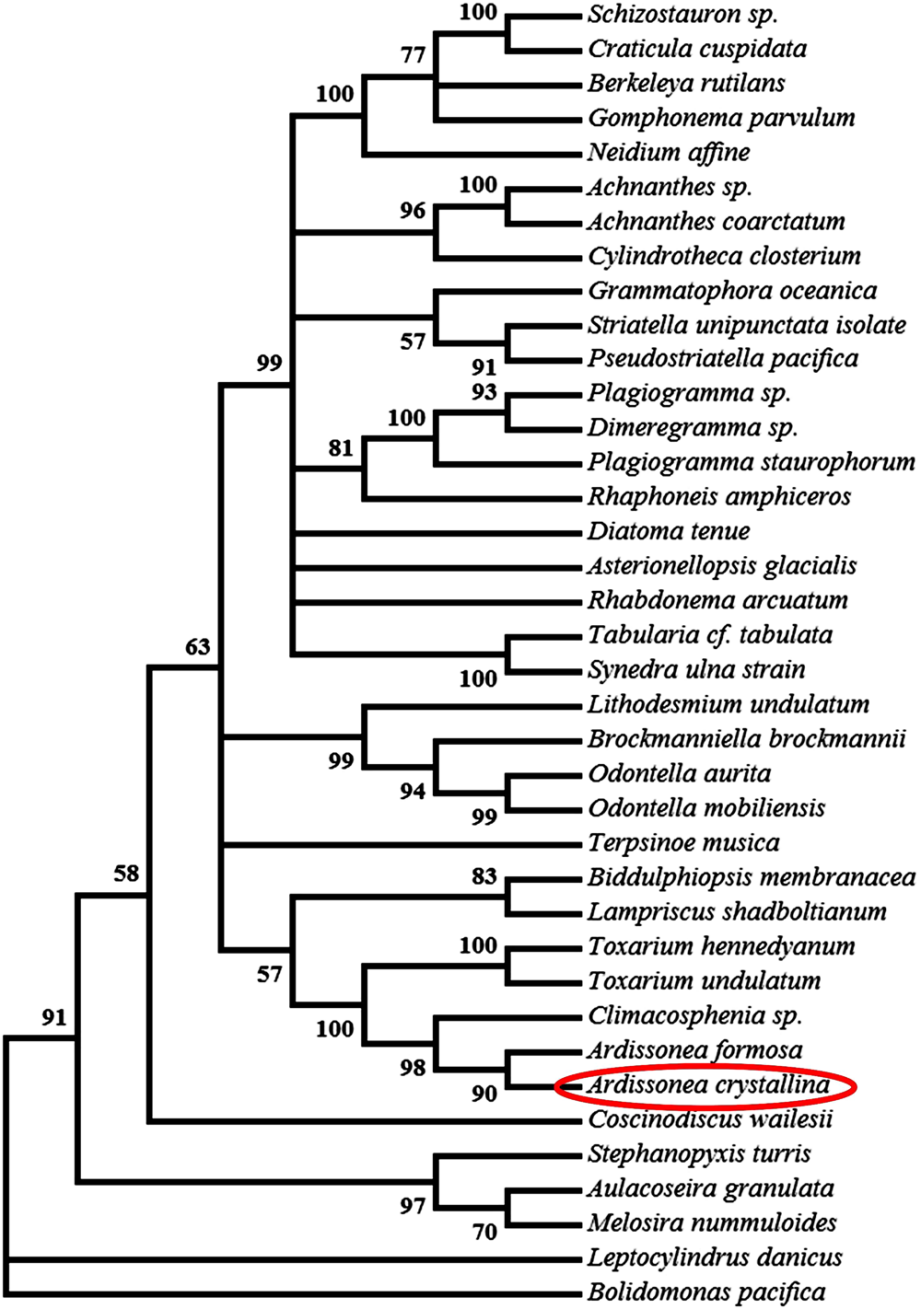
The three gene based (SSU, *rbc*L, *psb*C) phylogeny inferred by using the Maximum Likelihood method based on the Tamura-Nei model, conducted with 1000 bootstrap replications. The percentage of trees in which the associated taxa clustered together is shown next to the branches. *Bolidomonas pacifica* Guillou & Chrétiennot-Dinet was the out-group.

PCR amplification of the complete *rbc*L sequence was 1473 bp long (Supplementary Table S5, Supplementary Material). A BLAST search returned a 98% sequence identity (E-value 0.0) with *Ardissonea baculus* (AB430664). The SSU sequence retrieved from next-generation sequencing also linked *A. crystallina* with *A. baculus*, with 99% of identity and E-value 0.0 for 1775 bp long fragments. Maximum likelihood phylogenies using the Tamura-Nei model (3-genes set including SSU, *rbc*L, *psb*C) resulted in a tree unambiguously associating *A. crystallina* with other Toxariids (Fig. 10), in a clade containing *Toxarium hennedyanum, Toxarium undulatum*, *A. formosa* and *Climacosphenia* sp. with good support (bootstrap values ranging from 90 to 100). The multigene tree is presented as Supplementary material (Supplementary Fig. S4).

## Discussion

The pattern of the process of sexual reproduction found in *Ardissonea crystallina* is in many features unprecedented among centric diatoms. During gametogenesis, single gametes are produced by both male and female gametangia, usually with no residual bodies. No depauperating mitoses, common among centrics, were detected. Both male and female gametes keep all chloroplasts of the parental cells, and possibly pass both to the zygote. Although gametogenesis can be divided into two types — male and female — no flagellated gametes were observed here, in contrast to all centrics whose sexual reproduction has been examined to date. The mode of gametogenesis and valve patterning have been traditionally used as corner-stone characters supporting segregation of diatoms into centric and pennates. Four monoflagellated sperms are produced by a spermatocyte in centrics. In pennates, male gametogenesis results in only one or two non-flagellated gametes per meiocyte. Instead of flagella, locomotion of the *A. crystallina* male gametes resulted from sluggish amoeba-like movement accompanied with the production and retraction of thin cytoplasmic projections. The last facilitate search the female sex cells and motion of the male gametes to them. In general, the mechanism of locomotion of male gametes in *A. crystallina* is similar to that of the “core” araphid pennates *Pseudostaurosira trainorii* E. Morales^22^, *Tabularia fasciculata* (C.A. Agardh) Williams and Round, *T. tabulata* (C.A. Agardh) Snoeijs^23^, and *Ulnaria ulna* (Nitzsch) Enrenberg^24^, but more significantly, also to that of members of the “basal” araphids^25^. Remarkably, the mechanism of male gamete locomotion was a key diagnostic character in the recently proposed reclassification of the araphid pennates^26^. Thus, the course of male gametogenesis and behavior of male gametes in *A. crystallina* is more similar to those seen among pennates, not centrics.

The three gene based Maximum Likelihood phylogeny (Fig. 10) illustrates without any doubt that the studied strains of *A. crystallina* belong to the Toxariids group among polar centrics, and are clearly separated from the araphid pennate species. This position was also displayed by the multigene Maximum Likelihood phylogeny (Supplementary Fig. S4), whose strong bootstrap values undoubtedly associated Toxariids with *Lampriscus* species. It is worth noting that so far, the most closely-related species to *A. crystallina* whose auxosporulation has been studied in detail is *Hydrosera triquetra* Wallich, which undergoes a completely different kind of sexual reproduction than that seen in *Ardissonea*. In *Hydrosera*, as is typical of centrics, sexual reproduction is oogamous and involves flagellated sperm^27^. Auxospores have also been observed in an unidentified species of *Lampriscus* ^28^, a genus appearing to be the closest to the Toxariids in each molecular phylogeny^9,11,13,14,29^. Its pattern of gametogenesis has not been described. Apart from the closest neighbours to *A. crystallina* listed above, oogamous reproduction has been found in several other members of Mediophyceae: *Biddulphia* (“true” Biddulphia and some species transferred to *Odontella* and *Trieres*), *Odontella*, and *Cymatosira* ^2,30–32^. This suggests that the type of sexual reproduction found in *Ardissonea*, which is unusual in centric diatoms, is derived.

In most of the phylogenetic trees constructed so far, the Toxariids relate to the branch comprised of polar centric diatoms^5,8,9,11,12,33–35^ ^,present study^. In one case, however, *Ardissonea formosa* (Hantzsch) Grunow was found to be sister to araphid pennates^36^. Polar centric diatoms correspond to Clade 2a in early studies by Medlin and colleagues, and now recognized at the class level as the Mediophyceae^5,37–39^. In the phenogram inferred by Kooistra *et al*.^6^, *Toxarium undulatum* and the other sampled polar centrics were collapsed into a polytomy, thereby suggesting either the absence of a strict divergence between polar centrics and araphid pennates, or our inability to reliably distinguish which lineage among the polar centrics diverged first. Later papers with increased taxon sampling have appeared to better resolve the relationships between the polar centric lineages; in another phylogenetic tree^11^ *Toxarium* was sister to *Lampriscus* (bootstrap support = 72%), and with further taxon sampling^9^, not only were the Toxariids found to be monophyletic (bootstrap support = 88%), but the sister relationship to *Lampriscus* remained intact as well.

Medlin and Kaczmarska^5^ erected the new class Mediophyceae based in part on the order Mediales proposed by Russian scholars^40^, established to recognise the evolutionary significance of the polar centrics, thereby approaching a more natural evolutionary history of diatoms. The Pennales were assumed by Russian scholars to originate from ancient representatives of the Mediales, which was assumed to be an intermediate group. Recognition of the Mediophyceae has reignited and sharpened the discussion on separation of centric and pennate diatoms^9,41,42^. The question centres on significance of morphological duality. Which of the characters segregates diatoms in the most natural ways: valve patterning, sexual reproduction, auxospore structure or molecular phylogeny?

The closest relatives of the pennate diatoms remain unknown. There have been many molecular phylogenetic studies (comprehensively reviewed by Sorhannus & Fox^35^ and Medlin^38^) considering various diatom lineages as the possible sister group to the pennates. Among those *Toxarium*/Toxariids are repeatedly mentioned as a plausible candidate^9,33,39,43^, although other studies^8,35,44^ recovered the Cymatosirales as their sister group. Sorhannus and Fox^35^ found that *Toxarium undulatum* shares a strongly supported node with the Thalassiosirales. The investigated members of *Thalassiosira* (even as presently defined) demonstrated reproduction with formation of flagellated sperm (classical oogamy), typical of centric diatoms^45–51^. The single exception is *T. pseudonana* not exhibiting the size reduction-restitution cycle and hence no sexual reproduction and auxosporulation have been described in this species.

Some other authors have inferred the bipolar centric diatom *Attheya* lineage as sharing the most recent common ancestor with Bacillariophyceae^8,35,44^, but those were topologically distant from pennates in the analyses of the six chloroplast genes^15^. Sexual reproduction has been investigated in *A. decora*, and found to be oogamous^52^. Other polar centrics, including the Biddulphiopsids, *Lampriscus*, and some others (for details see ref.^9^) have also been considered as possible closest relatives to pennate diatoms.

Divergence between non-polar centric and polar diatoms is strongly suggested by the type and structure of their auxospores (e.g. refs^53,54^, except for Thalassiosirales). In radial centrics auxospores expand isometrically; polar centrics and pennates grow anisometrically because of specific auxospore walls containing either only incunabula or incanubula and perizonial bands, respectively. Radial and investigated polar centric diatoms demonstrate oogamous reproduction with eggs and flagellated sperm, whereas pennates are almost exclusively isogamous^2^, and none are known to produce flagellated sperm. Not a single exception has been recovered among 200 + pennate diatoms in which sexual reproduction has been investigated to date. “Spermatogenesis” described in *Pseudo-nitzschia multiseries* (Hasle) Hasle (as *Nitzschia pungens* Grunow f. *multiseries* Hasle^55,56^) was most likely an intercellular, flagellated parasite^57–59^, as further investigation in this species found sexual processes typical of pennates, with the absence of flagellated cells^60,61^.

Apart from the extremely elongated form, other morphological and behavioral traits emphasize the peculiarity of Toxariids amongst both centric and pennate diatoms. D. G. Mann noted^62^ (p. 26) that “there are also a few diatoms that have significantly different pattern centers, which may need to be recognized as further variants alongside the centric and pennate groups; these include *Toxarium, Synedrosphenia, Ardissonea* and *Climacosphenia*”. Interestingly, some cells of *A. crystallina* exhibit intricate patterns of silica deposition (see Fig. 1g,k), in some cases even resembling an annulus similar to that observed in the early stages of valve morphogenesis in another polar centric diatom, *Plagiogrammopsis vanheurckii* (Grunow) Hasle, von Stosch et Syvertsen^63^. This pattern, which in *A. crystallina* resembles chaotically arranged ribs, can hardly be considered a homologue of the annulus known in radial centrics. This disorganized patterning also appears to be somewhat rare, as we observed it in only a few completely developed frustules. A similar disorganized striation pattern in the valve center was also observed in another toxariid diatom, *A. fulgens* var*. gigantea* from Guam^64^.

A more speculative scenario might suppose that pennates and Toxariids have a more common ancestry than suggested by DNA sequence data. This scenario has roots in the conclusions inferred from the analysis of the evolution of meiotic patterns in oogenesis and spermatogenesis among centric diatoms^65,66^, which suggest that polar, not radial forms are primitive among centric diatoms. This, again, runs counter to all molecular phylogenies published on diatoms, which unilaterally support the notion that it is the radial (non-polar) centrics that represent “primitive” forms. Whereas the results of a cladistic analysis^67^ suggested that at least the elongate outline of *Toxarium* was not a homoplasy, but may be synapomorphic for a larger, more inclusive clade than the traditional Pennales; this idea was disputed in Medlin *et al*.^12^.

A unique mode of movement was described in vegetative cells of *Toxarium undulatum* Bailey; they could move being in near vertical position at speeds of up to 4 µm·s^−1^ through secretion of mucilage from the cell poles^11^. We observed the same manner of movement in *A. crystallina*, and as far as we are aware, this type of motility has not been observed in any other member of polar centric or pennate diatoms.

The present work also underlines the need for further inquiries into the sexual reproduction of the Toxariids. The auxosporulation of *Toxarium*, *Climacosphenia* or any other species of *Ardissonea* has yet to be observed. Accordingly, there is no indication that the peculiar modes of gametogenesis and auxosporulation found in *A. crystallina* are restricted to this single species, common to the whole genus, or shared with the other genera of Toxariids. The importance of documenting this mode of auxosporulation in the Toxariids is twofold. First, knowing the systematic breadth of *A. crystallina*’s type of gametogenesis will help in using molecular phylogenetic dating studies to indicate its time of appearance. For example, Sorhannus^29^ calculated, using small subunit ribosomal RNA sequence divergence, that the *Lampriscus-Toxarium* lineage diverged from the other centric diatoms some 80 Ma. Considering the limited reproductive structures that have been observed in *Lampriscus* ^28^, the pennate-like mode of auxosporulation documented in *A. crystallina* should have developed more recently than the *Lampriscus* + *Toxarium* split. Secondly, the Toxariids could provide us with an ideal platform to study the molecular basis of the pennate-like modes of gametogenesis and auxosporulation. As molecular evidence suggests the position of Toxariids within the polar centric diatoms is a derived lineage, all of their closest genetic ancestors are likely to have a more oogamous reproductive type. This makes the Toxariids an attractive model for comparative genomic or transcriptomic studies, as the relatively short evolutionary distance between the Toxariids and their more “classically centric” sister taxa is likely to limit the overall genomic differences to valve morphology and reproductive characters. In this model, we can study the molecular and genetic differences between “pennate” and “centric” diatoms without knowing the closest ancestor to pennates, using the Toxariids as a proxy for pennate-like characters, such as the *A. crystallina* form of gametogenesis.

In conclusion, the class Mediophyceae represents polar centric diatoms whose auxospore envelope structure tends to be more similar to pennates than to radial centrics^8,68^. Regarding the mode of sexual reproduction, however, all previously-studied Mediophycean taxa exhibited the more classically “centric” oogamy. The mode of reproduction discovered in *Ardissonea crystallina*, without flagellated gametes, and characteristic motion of the male gametes capable of forming temporary cytoplasmic projections are much more similar to that exhibited by certain araphid pennate diatoms, including members of the “basal” araphids^25^. This suggests either a loss of flagellate sperms in *Ardissonea* independent from this in pennates (homoplasy), or a close ancestral relationship between *Ardissonea* (and possibly other Toxariids) and pennate diatoms. The latter assumption seems unlikely in view of the considerable amount of molecular phylogenetic data suggesting the Toxariids belong to the polar centric diatoms, though we cannot rule out the position of the Toxariids as a sister lineage to the pennates, because at this time the sister group of the pennate diatoms remains unresolved^15,38^. It is clear now that if we wish to clarify the evolutionary history of Toxariids the investigation should be focused on the structure and development of the auxospore and the mode of initial cell morphogenesis, not only within the Toxariids, but also in other closely-related Mediophycean taxa, such as *Lampriscus*, *Trigonium* and *Biddulphiopsis*. We would also propose that the Toxariids and their relatives in the Mediophyceae provide an excellent model for investigation of the evolution of sex determination systems.

## Materials and Methods

### Establishment of clonal cultures

A natural population of *Ardissonea crystallina* is located in the Bay Kazachya of the Black Sea (Sebastopol, Russia). Samples of benthos and periphyton were collected at a depth of about 0.4 m from a site with geographical coordinates 44°34′19″N, 33°24′07″E in July and November 2011, April 2012, January and June 2014, and February 2015. Samples were delivered to the laboratory and added to culture media. Single cells were isolated into clonal cultures with the aid of Pasteur micropipettes. In total, 30 clonal cultures were established. The clones were designated as Y.MM.DD–N, where Y is the last digit of the year, MM – month, DD – day of isolation, N – short name of the clone. Clonal cultures used for mating experiments were not axenic; they might have contained bacteria and small flagellates. Cultures were grown in artificial seawater prepared according to the ESAW recipe^69^ with small modifications (addition of FeNH_4_-citrate and reduced amount of NaH_2_PO_4_ and NaNO_3_) and diluted to a salinity of *ca*. 20‰ with distilled water. Cultures were maintained in exponential growth phase through periodical (once a week) transfer into fresh growth media. Glass culture vessels (200 ml Erlenmeyer flasks or 9 cm Petri dishes) were placed in a thermo-stabilized room at 20 °C under natural light from a north-facing window. Voucher specimens of the clones (fixed in 50% alcohol) are kept in the algae and microbiota laboratory at the Karadag scientific station. Several live clones are continuously maintained in the culture collection^70^.

### Mating experiments

On the fifth to sixth day after reinoculation into fresh medium, exponentially growing cultures were mixed to initiate heterothallic sexual reproduction. For sexual reproduction to be successful two main criteria must be met: appropriate apical cell length, which must be below the critical threshold, and availability of a compatible partner in the case of heterothallic reproduction. Pairwise mixtures of clones were made in 5 cm glass Petri dishes and cultures were then inspected daily using a microscope to check for the occurrence of sexual cells. Homothallic reproduction was also observed in some monoclonal cultures. No change in culture conditions was required to induce both intraclonal (homothallic, *sensu* Kaczmarska *et al*.^71^, p. 11) and interclonal (heterothallic) sexual reproduction.

### Light and electron microscopy

LM observations were made using a Biolar PI (PZO, Poland) microscope with differential interference contrast (DIC) optics and a NIB-100 inverted microscope (China). A Canon PowerShot A640 digital camera with a 1/1.8″ CCD-type sensor with 10·10^6^ effective pixels, was used to photograph live stages. The full-size images were acquired at a resolution of 2816 × 2112 pixels. For SEM examination, diatom cells were cleaned of organic material by boiling in 35% hydrogen peroxide (H_2_O_2_), the cell suspension was then centrifuged and rinsed with distilled water several times, pipetted onto aluminium stubs, air-dried and sputter-coated with gold. Electron micrographs were acquired using a JEOL JSM-5600 scanning electron microscope. Image contrast and brightness were adjusted by using ACDSee 5.0 software (https://www.acdsee.com/en/index).

Digitally acquired LM and SEM images were used for cell measurements: length, width, etc., using ImageJ software (http://rsb.info.nih.gov/ij/). In addition, several measurements of cells were taken under a microscope equipped with an ocular ruler calibrated by using a standard stage micrometer.

DAPI (4′,6-diamidino-2-phenylindole) staining of the nuclei was fulfilled according to the Protocol for Fluorescence Imaging https://www.thermofisher.com/ua/en/home/references/protocols/cell-and-tissue-analysis/protocols/dapi-imaging-protocol.html). Images were captured using a LIF-302 inverted fluorescence microscope (Leader Precision Instrument Co., Ltd, China). Additional DAPI epifluorescence and brightfield image pairs were acquired from glutaraldehyde fixed (2.5% v/v) material using a Zeiss Axioskop 2 plus upright microscope (Carl Zeiss, Oberkochen, Germany) with AxioCam HR color camera (1300 × 1030 pixels), HBO 100 mercury vapour illumination source and epifluorescence filter set 01. Brightfield images were converted to grayscale for presentation. Wet mounts were stained with DAPI-containing Vectashield mounting medium (Vector Laboratories, Burlingame, CA, USA) as per manufacturer’s instructions. Cell measurements were performed using dmfMeasure^72^. RGB and grayscale values for the entire field of acquired images were adjusted for optimum contrast and brightness using the “Levels” function in Adobe Photoshop CC 2017 (Adobe Systems Incorporated, San Jose, CA, USA).

### Molecular species identification and phylogenetic analyses

The clone 3.0722-E was cultivated under the same conditions as described above, and harvested in mid-exponential phase. Pellets of cells were generated by gentle centrifugation at 900 × g for 10 minutes. Fresh material was crushed in liquid nitrogen with a mortar and pestle, and DNA was extracted following the Doyle and Doyle protocol^73^. The primers used for amplification were DP*rbc*L1 and DP*rbc*L7^74^ modified according to Jones *et al*.^75^. PCR amplifications were conducted using an Eppendorf Mastercycler Gradient in a final volume of 25 µL, with 0.2 mM primers 2.5 U GoTaq® Flexi DNA Polymerase (Promega), 1xgreen GoTaq® flexi buffer, 2.5 mM MgCl_2_, 200 mM PCR nucleotide mix (Promega, France). Thirty-six cycles were conducted as follows: initial denaturing (94 °C, 1 min), annealing (52 °C, 1 min), extension (72 °C, 1 min 30 s).

After completion of the PCR reaction, products were separated by agarose gel electrophoresis and confirmed visually using a CN-1000 Darkroom from Vilber Lourmat. Bands of the expected size were picked and eluted with a Wizard® SV Gel and PCR Clean-up System (Promega). Eluted PCR products were integrated into pGEM® T-Easy Vector (Promega). Competent *Escherichia coli* cells NEB-5-alpha from New-England Biolabs (Evry, France) were transformed with the recombined vector. A white/blue selection helped selection of positive colonies. Sequencing reactions were thereafter provided by Beckmann Coulter Genomics (UK, http://www.cogenicsonline.com) using universal SP6 and T7 primers. The same clone was later cultivated under the same conditions, and its DNA was extracted the same way. It was then sequenced by BGI Shenzhen on an Illumina Hiseq. 4000, 300 bp insert and 150 PE sequencing, with 3 Gb of clean data. Data were assembled using Ray 2.3.0^76^, with k-mer 31 to 45. Identification and annotation of the chloroplastic genes and the SSU were performed using a set of automated tools developed in Laval University^77^.

### Phylogeny

For the three gene based (SSU, *rbc*L, *psb*C) phylogenetic tree (Fig. 10), a set of sequences from various species of diatoms (Supplementary Table S6, Supplementary Material) was downloaded from GenBank database (https://www.ncbi.nlm.nih.gov/genbank/). They were aligned with the corresponding sequences obtained from *A. crystallina* (SSU, *rbc*L, *psb*C, respectively deposited as MF555728, MF578744, MF578745) using Mega 6 software^78^. After trimming, the same software was used for Maximum Likelihood phylogeny, which was conducted with 1000 bootstrap replications, a cut-off value of 50% and the tree was rooted with sequences from *Bolidomonas pacifica*.

For the multigene phylogeny, the pre-existing alignment^44^ was downloaded from the deposit website http://datadryad.org/resource/doi:10.5061/dryad.610md/1. Concatenated sequences of the corresponding genes from *A. crystallina* were added (SSU, *atp*B, *psa*B, *psa*A, *psb*A, *psb*C, *rbc*L, respectively deposited as MF555728, MF578740, MF578741, MF578742, MF578743, MF578744, MF578745). Alignments were done using Clustal Omega^79^ and Maximum Likelihood phylogeny was conducted with RAxML 8 with 100 bootstrap replications^80^. The resulting tree was rooted with *B. pacifica*, and a cut-off value of 50% was applied

### Data Availability

All data analysed or generated during this study are included in this published article (and its Supplementary Information files). Additional information is available from the authors upon request. All new gene sequences obtained from *A. crystallina* were deposited in Genbank database (https://www.ncbi.nlm.nih.gov/genbank/) with the accession numbers MF555728, MF578740, MF578741, MF578742, MF578743, MF578744, MF578745 for SSU, *atp*B, *psa*B, *psa*A, *psb*A, *psb*C, and *rbc*L genes respectively.

## Electronic Supplementary Material

Supplementary information (Davidovich et al.)
