# Supplementary material for: Ardissonea crystallina has a type of sexual reproduction that is unusual for centric diatoms
Source: Sci Rep. 2017 Nov 7;7:14670. doi: 10.1038/s41598-017-15301-z (PMC5676960; doi:10.1038/s41598-017-15301-z)
Supplement: Supplementary file 1 — Supplementary information (Davidovich et al.) [file 41598_2017_15301_MOESM1_ESM.pdf]

Ardissonea crystallina has a type of sexual reproduction that is unusual for centric diatoms

Nickolai A. Davidovich, Olga I. Davidovich, Yulia A. Podunay, Romain Gastineau, Irena Kaczmarska, Aloisie Poulíčková, and Andrzej Witkowski

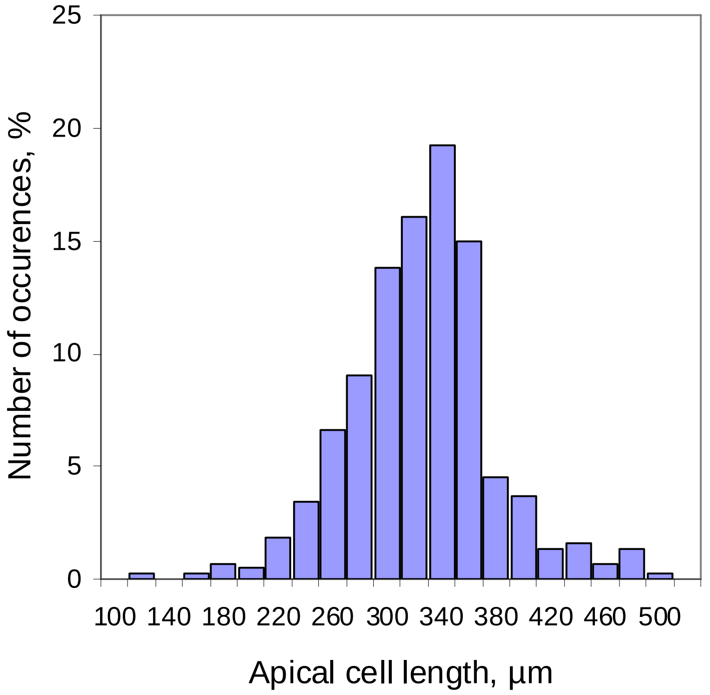

Supplementary Fig. S1. Cell length distribution in the native population of *Ardissonea crystallina* (C.Agardh) Grunow inhabiting Bukhta Kazachya, the Black Sea; sampling on July 2011, the number of cells measured *N*=441. The distribution is normal with a confidence of 77.76% (Anderson-Darling normality test)

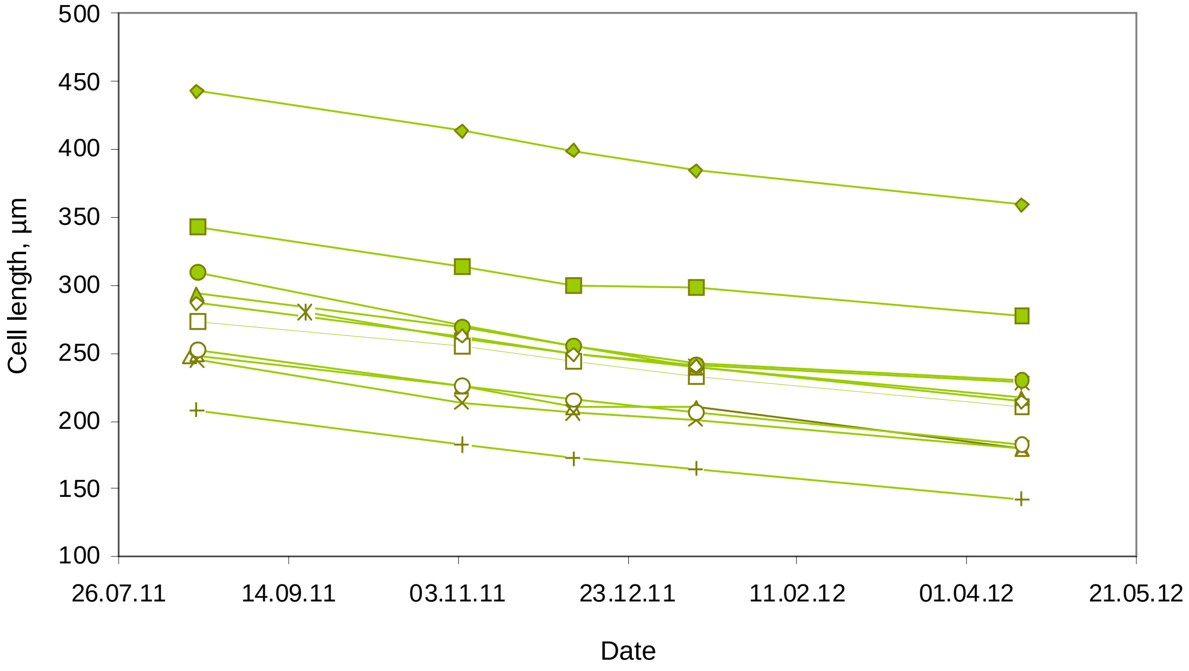

Supplementary Fig. S2. Decrease of the apical cell length in *Ardissonea crystallina* (C.Agardh) Grunow clones growing in cultures

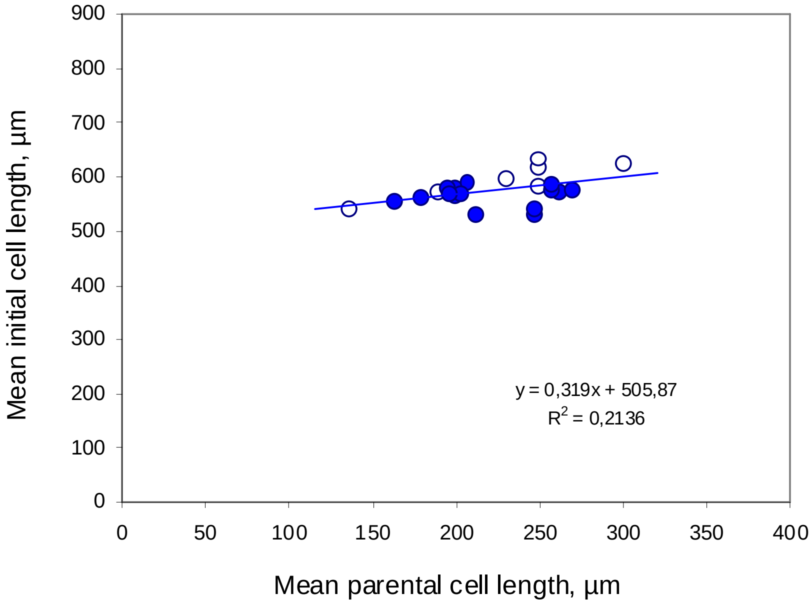

Supplementary Fig. S3. Dependence between parental and initial cell length in *Ardissonea crystallina*. Shade circles correspond to the initial cells resulted from interbreeding, empty circles indicate intraclonal reproduction

Supplementary Figure S4. Maximum likelihood tree obtained from concatenated sequences (including the genes SSU, *atp*B, *psa*A, *psa*B, *psb*A, *psb*C, *rbc*L) representing 208 species of diatoms including *Ardissonea crystallina*. Corresponding sequences from *Bolidomonas pacifica* are being used as an outgroup. Bootstrap values for 100 bootstrap replicates, with a cut-off of 50% applied

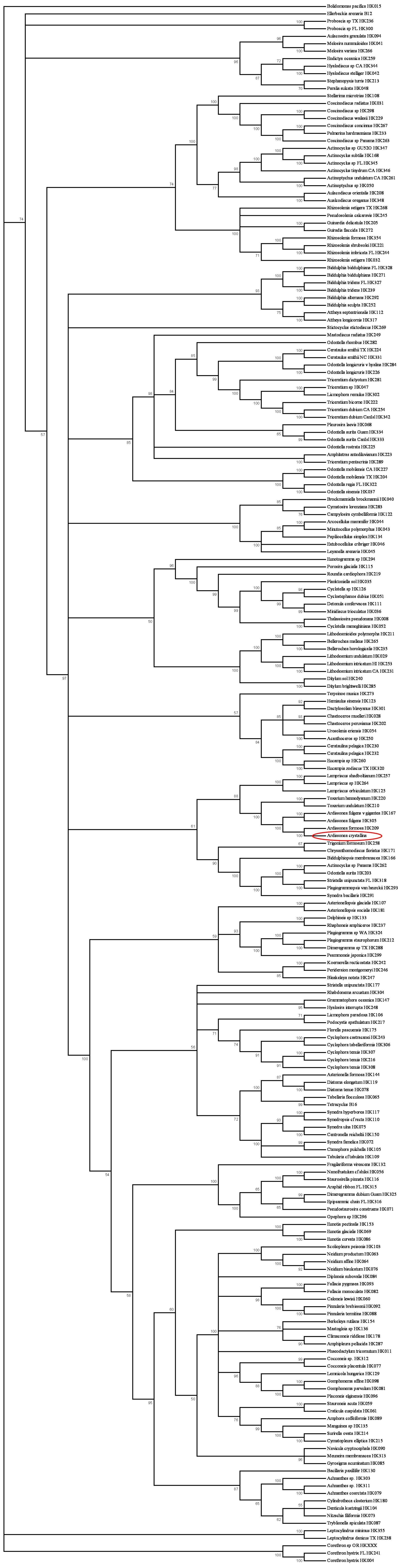

Supplementary Table S1. The apical length of cells in *Ardissonea crystallina* clones derived from natural population and tested in mating experiments*

Clone name	Date of measuring	Cell length, μm				
		**Mean**	SE	***N***	Min	Max
**1.0721-D**	26.08.2011	**307.2**	6.0	**20**	270	339
	02.11.2011	**316.6**	1.2	**12**	311	325
	28.11.2011	**307.2**	2.8	**5**	298	311
**1.0721-F**	04.11.2011	**260.2**	1.8	**10**	249	270
	07.12.2011	**249.1**	1.5	**10**	242	256
	12.01.2012	**240.8**	1.7	**10**	235	249
	17.04.2012	**228.4**	2.1	**10**	215	235
**1.0721-K**	27.04.2015	**154.3**	1.1	**10**	152	159
**1.0721-Q**	16.08.2011	**247.7**	1.1	**10**	242	253
	18.08.2011	**248.8**	1.0	**11**	242	256
	04.11.2011	**225.6**	1.5	**10**	221	235
	07.12.2011	**210.4**	1.1	**10**	208	215
	12.01.2012	**210.4**	1.1	**10**	208	215
	17.04.2012	**179.9**	1.8	**10**	173	187
**1.0721-R**	18.08.2011	**310.1**	1.3	**11**	304	318
	04.11.2011	**269.9**	1.8	**10**	263	277
	07.12.2011	**255.3**	2.8	**10**	242	270
	12.01.2012	**242.2**	2.1	**10**	235	256
	17.04.2012	**230.4**	1.8	**10**	221	235
**1.0721-V**	18.08.2011	**274.0**	1.0	**11**	270	277
	04.11.2011	**255.3**	2.8	**10**	242	277
	07.12.2011	**244.3**	1.8	**10**	235	256
	12.01.2012	**233.2**	1.5	**10**	228	242
	17.04.2012	**211.1**	1.2	**10**	208	215
**1.0721-W**	18.08.2011	**245.0**	1.1	**10**	242	249
	04.11.2011	**213.8**	2.6	**10**	201	221
	07.12.2011	**206.2**	2.9	**10**	194	221
	12.01.2012	**201.4**	1.6	**10**	194	208
	17.04.2012	**179.9**	1.8	**10**	173	187
**1.0725-I**	02.11.2011	**290.6**	2.3	**12**	270	298
**1.0725-J**	26.08.2011	**328.4**	7.8	**20**	284	367
	02.11.2011	**336.2**	1.8	**12**	325	346
**1.0725-K**	26.08.2011	**307.6**	3.1	**20**	284	325
	04.11.2011	**267.8**	2.1	**10**	256	277
	28.11.2011	**273.3**	4.3	**8**	249	284
**1.0725-S**	18.08.2011	**293.8**	1.1	**11**	291	298
	04.11.2011	**268.5**	2.3	**10**	263	284
	07.12.2011	**254.7**	1.7	**10**	249	263
	12.01.2012	**239.4**	1.8	**10**	228	249
	17.04.2012	**217.0**	1.1	**11**	215	221
**1.0725-T**	18.08.2011	**252.9**	1.1	**11**	249	256
	04.11.2011	**226.3**	1.5	**10**	221	235
	07.12.2011	**215.9**	1.7	**10**	208	228
	12.01.2012	**206.2**	3.4	**10**	180	215
	17.04.2012	**183.0**	1.7	**9**	173	187
**1.0725-X**	18.08.2011	**208.3**	1.2	**10**	201	215
	04.11.2011	**182.7**	1.1	**10**	180	187
	28.11.2011	**170.2**	2.8	**5**	166	180
	28.11.2011	**169.5**	2.0	**4**	166	173
	07.12.2011	**172.3**	1.2	**10**	166	180
	12.01.2012	**164.7**	1.7	**10**	159	173
	17.04.2012	**142.6**	1.5	**10**	138	152
**1.0727-A**	26.08.2011	**387.5**	15.5	**20**	318	464
	02.11.2011	**294.1**	1.0	**12**	291	298
	29.04.2015	**87.4**	1.4	**11**	76	90
**1.0727-Y**	18.08.2011	**287.2**	1.2	**10**	284	291
	04.11.2011	**262.3**	1.6	**10**	256	270

	07.12.2011	**249.1**	1.8	**10**	242	256
	12.01.2012	**240.1**	2.1	**10**	228	249
	17.04.2012	**214.5**	2.1	**10**	208	228
**1.0801-G**	26.08.2011	**384.1**	16.3	**20**	304	464
	02.11.2011	**430.2**	1.2	**12**	422	436
**1.0801-N**	02.11.2011	**291.2**	2.9	**12**	270	304
	27.04.2015	**147.4**	1.1	**10**	145	152
**1.0801-Z**	18.08.2011	**343.5**	1.1	**11**	339	346
	04.11.2011	**314.2**	2.1	**10**	304	325
	07.12.2011	**300.3**	1.5	**10**	291	304
	12.01.2012	**298.9**	2.5	**10**	291	311
	17.04.2012	**278.2**	1.7	**10**	270	291
**1.0803-B**	15.09.2011	**295.1**	4.4	**20**	270	318
	02.11.2011	**297.6**	1.9	**12**	284	304
**1.0808-B**	19.09.2011	**316.1**	1.1	**22**	304	325
	19.09.2011	**300.2**	4.0	**24**	270	325
	02.11.2011	**299.9**	1.3	**12**	291	304
**3.0711-C**	22.01.2014	**236.7**	1.4	**10**	228	242
	30.06.2014	**209.5**	1.9	**11**	201	215
	25.02.2015	**214.5**	1.3	**11**	208	221
	24.04.2015	**174.4**	4.4	**10**	145	187
	29.04.2015	**174.4**	3.7	**10**	152	194
	24.02.2016	**182.7**	1.1	**10**	180	187
**3.0722-A**	30.06.2014	**208.2**	3.7	**11**	180	221
	25.02.2015	**189.4**	1.1	**11**	187	194
	27.04.2015	**169.5**	2.1	**10**	159	180
	30.07.2015	**134.9**	1.2	**10**	131	138
	12.10.2015	**127.4**	2.2	**12**	118	138
**3.0722-D**	20.01.2015	**241.6**	9.1	**12**	208	277
	29.04.2015	**246.8**	11.4	**12**	187	284
	13.07.1015	**235.3**	1.0	**10**	228	242
	12.10.2015	**222.0**	1.3	**12**	215	228
**3.0722-E**	22.01.2014	**236.7**	0.9	**10**	235	242
	30.06.2014	**216.4**	1.0	**11**	215	221
	25.02.2015	**222.7**	4.8	**11**	187	235
	30.07.2015	**190.3**	1.2	**10**	187	194
**3.0726-A**	22.01.2014	**201.4**	1.2	**10**	194	208
	30.06.2014	**172.4**	1.5	**11**	166	180
	25.02.2015	**168.0**	1.0	**11**	166	173
	27.04.2015	**124.6**	0.0	**10**	125	125
	13.05.2014	**188.1**	1.3	**11**	180	194
**3.0726-C**	22.01.2014	**201.4**	1.2	**10**	194	208
	25.02.2015	**176.1**	1.1	**11**	173	180
	13.07.1015	**137.8**	2.0	**12**	125	145
**3.0726-D**	22.01.2014	**337.7**	1.4	**10**	332	346
	30.06.2014	**303.2**	1.8	**11**	298	311
	25.02.2015	**303.2**	3.7	**11**	277	318
	13.07.1015	**248.5**	1.7	**11**	242	256
**3.0726-E**	22.01.2014	**332.9**	1.2	**10**	325	339
	30.06.2014	**304.5**	1.3	**11**	298	311
	25.02.2015	**273.0**	3.8	**11**	249	291
	29.04.2015	**262.3**	1.6	**10**	256	270
	13.07.1015	**219.6**	1.6	**11**	215	228

Note. SE, standard error; *N*, number of cells measured; Min and Max, minimal and maximal values accordingly; blue and red colour of the clone names corresponds to male and female mating types accordingly; *, the maximal apical length of cells able to enter sexual reproduction was equal 304 μm

Supplementary Table S2. The apical length of initial cells in *Ardissonea crystallina* resulted from hetero- and homothallic sexual reproduction

Clone name	Date of measuring	Cell length, μm				
		**Mean**	SE	***N***	Min	Max
**Heterothallic reproduction in the mixture of two clones**						
**(1.0721-F + 1.0721-V)**	07.12.2011	**541,1**	7,6	**15**	484	574
**(1.0721-W + 1.0725-T)**	07.12.2011	**533,2**	7,8	**20**	477	595
**(1.0725-S + 1.0721-W)**	02.11.2011	**576,4**	6,5	**10**	547	609
**(1.0725-T + 1.0721-V)**	26.09.2011	**574,4**	9,0	**5**	554	602
**(1.0727-Y + 1.0721-V)**	07.12.2011	**530,8**	8,5	**20**	457	588
**(3.0722-A + 3.0711-C)**	23.01.2014	**582,7**	11,0	**5**	554	609
**(3.0722-A + 3.0711-C)**	02.03.2015	**570,0**	5,6	**11**	547	595
**(3.0722-A + 3.0722-E)**	23.01.2014	**568,1**	5,8	**11**	540	602
**(3.0722-A + 3.0722-E)**	30.06.2014	**577,5**	3,5	**11**	554	588
**(3.0722-A + 3.0722-E)**	02.03.2015	**590,1**	3,6	**11**	574	602
**(3.0722-A + 3.0722-E)**	30.07.2015	**556,7**	3,7	**9**	540	574
**(3.0722-A + 3.0726-E)**	30.06.2014	**589,5**	3,5	**11**	574	602
**(3.0726-A + 3.0722-E)**	30.06.2014	**580,0**	2,3	**11**	574	595
**(3.0726-A + 3.0722-E)**	02.03.2015	**571,2**	4,7	**11**	547	602
**(3.0726-C + 3.0726-E)**	20.07.2015	**563,7**	7,5	**15**	505	595
**Homothallic reproduction in the clone**						
**(1.0721-Q) intra +**	16.08.2011	**619,3**	17,3	**2**	602	637
**(1.0721-Q) intra +**	18.08.2011	**634,9**	4,4	**4**	623	644
**(1.0721-Q) intra +**	26.09.2011	**584,9**	9,5	**19**	519	678
**(1.0721-W) intra +**	02.11.2011	**597,2**	5,7	**20**	561	650
**(1.0808-B) intra +**	30.11.2011	**627,4**	11,1	**6**	602	671
**(3.0722-A) intra +**	30.07.2015	**543,9**	6,1	**10**	512	574
**(3.0726-A) intra +**	13.05.2014	**573,8**	6,8	**12**	547	602

Note. SE, standard error; *N*, number of cells measured; Min and Max, minimal and maximal values accordingly; blue and red colour of the clone names corresponds to male and female mating types accordingly

Supplementary Table S3. Cardinal cell length in the life cycle of *Ardissonea crystallina*

	Apical cell length, μm		Number of cells measured
	min	max	
Vegetative cells	76	678	2987
Gametangia	113	304	294
Initial cells	400	678	270

Supplementary Table S4. Mating compatibility between *Ardissonea crystallina* clones

Clone	Morphological sex	Mating type	1.0721-D	1.0721-F	1.0721-K	1.0721-Q	1.0721-R	1.0721-V	1.0721-W	1.0725-I	1.0725-J	1.0725-K	1.0725-S	1.0725-T	1.0725-X	1.0727-A	1.0727-Y	1.0801-G	1.0801-N	1.0801-Z	1.0803-B	1.0808-B	3.0722-A	3.0722-E	3.0711-C	3.0722-A	3.0722-B	3.0722-D	3.0722-E	3.0726-A	3.0726-C	3.0726-D	3.0726-E
		Mating type	mt1	mt1	mt1	mt2	mt1	mt2	mt2	mt2	mt2	mt1	mt1	mt1	mt2	mt2	mt1	mt2	mt2	mt1	mt1	mt2	mt2	mt1	mt1	mt2	mt1	mt2	mt1	mt2	mt2	mt2	mt1
1.0721-D	M	mt1																															
1.0721-F		mt1																															
1.0721-K		mt1																															
1.0721-Q		mt2	3	2	2	2																											
1.0721-R		mt1		0																													
1.0721-V		mt2	1	3	2		2	1																									
1.0721-W		mt2		3			3	1	2																								
1.0725-I		mt2	2							1																							
1.0725-J		mt2	2					0																									
1.0725-K	M	mt1						3		2	1																						
1.0725-S		mt1					0	3	2																								
1.0725-T		mt1		0	0		0	2	2		1	0																					
1.0725-X	F	mt2	2	2			2	0	0	0	0	2	2	2	2																		
1.0727-A		mt2	2		2							1			0	1																	
1.0727-Y		mt1		0	0		0	3					0	0	2																		
1.0801-G		mt2	2																														
1.0801-N		mt2	2	3											0		3																
1.0801-Z		mt1		0				2			0	0	0	0	2	0	0		3														
1.0803-B		mt1	2												0																		
1.0808-B		mt2	2		3																	2											
3.0722-A		mt2						0			0	2																					
3.0722-E		mt1						2			2																						
3.0711-C	M	mt1																															
3.0722-A	F	mt2																							3	1							
3.0722-B	M	mt1																							0	3							
3.0722-D		mt2														0												1					
3.0722-E	M	mt1																							2	3	0						
3.0726-A	F	mt2																							3	2	2		3	3			
3.0726-C		mt2																							2	0	1		2	0			
3.0726-D		mt2																							2	0	1		2			1	
3.0726-E	M	mt1														2			2						0	3	0	2	0	3	3	1	3

Numbers indicate maximal score of the auxosporulation abundance, 0 – no auxospores, 1- a few auxospores per Petri dish, 2 – auxospores not rare, 3 – plentiful auxosporulation; empty cells – results were not obtained. Underlined are clones capable of intraclonal sexual reproduction. "**M**" and "**F**" are morphologically defined sexes producing active and passive gametes, respectively. Mating types were conveniently designated as **mt1** (Male) and **mt2** (Female)

Supplementary Table S5.

>*Ardissonea crystallina* rbcL sequence

ATGTCTCAAGCTGTATCAGAACGTACTCTAATTAAAAGTGAGCGTTATGAATCTGGTGTAATTCCATATGC
TAAGATGGGTTACTGGGATGCTTCATATTCAGTAAAAACTACTGATATTCTTGCTTTATTCCGTATTACACC
ACAACCAGGCGTAGATCCTGTAGAAGCAGCTGCAGCAGTAGCAGGTGAATCATCTACAGCTACATGGAC
GGTTGTTTGGACAGATTTATTAACGGCATGTGATCGTTATCGTGCTAAAGCTTACCGTGTAGATCCAGTA
CCGAATGCAACTGACCAATATTTTGCATTTGTTGCATACGAATGTGATTTATTTGAAGAAGGTTCTCTTCC
GAACTTAACAGCTTCTATTATTGGTAACGTTTTTGGTTTCAAAGCCATTTCAGCGTTACGTTTAGAAGATA
TGCGTATTCCATTTGCATATTTAAAAACATTCCAAGGTCCTGCTACAGGTGTAGTTGTAGAACGTGAACGT
TTAAATAAATATGGTGCTCCATTATTAGGTGCGACTGTAAAACCTAAATTAGGTCTTTCAGGCAAAAACTA
TGGACGTGTAGTATATGAAGGTTTAAGAGGTGGTTTAGATTTCTTAAAAGATGATGAAAACATTAATTCT
CAACCATTCATGCGTTGGAGAGAACGTTACTTATACGTAATGGAAGGTATTAACCGTGCTGCAGCCTCAA
CAGGTGAAGTTAAAGGTTCATACTTAAATATTACAGCGGCAACAATGGATGAAGTAATTTTACGTGCTGA
ATTTGCTAAACAATTAGGTTCAGTTATTGTAATGATTGATTTAGTATTAGGTTATACAGCAATTCAAAGTG
CTGCAATTTGGGCACGTGAAAATGACATGATTTTACATTTACATCGTGCAGGTAACTCTACTTACGCTCGC
CAAAAAAATCATGGTATTAACTTCCGTGTTATTTGTAAATGGATGCGTATGTCAGGTGTAGATCATATCCA
CGCTGGTACAGTAGTAGGTAAATTAGAAGGTGATCCTTTAATGATTAAAGGTTTCTATGATACTTTATTAT
TAACAACATTAAAAGTTAACTTACCATACGGTATTTTCTTTGATATGGATTGGGTTAGTTTACGTAAATGT
CTTCCAGTTGCATCTGGTGGTATTCATTGTGGTCAAATGCACCAATTAGTTCATTATTTAGGTGATGATGT
TGTATTACAATTTGGTGGTGGTACTATTGGTCACCCTGATGGTATTCAAGCTGGTGCTACAGCTAACCGT
GTTGCATTAGAATCTATGATTTTAGCTCGTAACGAAGGTGCTGATTACTTTAATGATGAAGTAGGTCCTAA
AATCTTACGTGATGCTGCGAAAACTTGTGGTCCATTACAAACAGCATTAGATTTATGGAAAGATATTAGTT
TTAACTATACTTCAACAGATACTTCTGATTTCTCTGAAACACCAACAGCAAACGTATAA

Supplementary Table S6. Species, strain identifiers and GenBank accession numbers used in the three gene based (SSU, rbcL, psbC) phylogenetic tree

Species & strain identifier	SSU	*rbc*L	*psb*C
Achnanthes coarctatum strain UTEX FD185	HQ912594	HQ912458	HQ912287
Achnanthes sp. SZCZCH113	KT943601	KT943615	KT943626
Ardissonea crystallina clone 3.0722-E	MF555728	MF578744	MF578745
Ardissonea formosa strain ECT3655	HQ912653	HQ912517	HQ912346
Asterionellopsis glacialis strain CCMP134	HQ912613	HQ912477	HQ912306
Aulacoseira granulata strain UTEX FD301	HQ912606	HQ912470	HQ912299
Berkeleya rutilans strain ECT3616	HQ912637	HQ912501	HQ912330
Biddulphiopsis membranacea strain ECT3655	HQ912638	HQ912502	HQ912331
Bolidomonas pacifica strain CCMP1866	HQ912557	HQ912421	HQ912251
Brockmanniella brockmannii strain CCMP151	HQ912565	HQ912429	HQ912259
Climacosphenia sp. ECT3758	HQ912685	HQ912549	HQ912371
Coscinodiscus wailesii strain ECT3847	HQ912668	HQ912532	HQ912360
Craticula cuspidata strain UTEX FD35	HQ912581	HQ912445	HQ912274
Cylindrotheca closterium strain CCMP1855	HQ912645	HQ912509	HQ912338
Diatoma tenue strain UTEX FD106	HQ912593	HQ912457	HQ912286
Dimeregramma sp. SZCZP256	KR048190	KR048210	KT119340
Gomphonema parvulum strain UTEX FD241	HQ912595	HQ912459	HQ912288
Grammatophora oceanica strain CCMP410	HQ912634	HQ912498	HQ912327
Lampriscus shadboltianum isolate Pan-1	KC309497	KC309571	KC309644
Leptocylindrus danicus strain SZN-B650	KC814808	KC814842	KC814848
Lithodesmium undulatum strain CCMP1806	DQ514846	DQ514765	DQ514683
Melosira nummuloides strain CCMP482	HQ912566	HQ912430	HQ912260
Neidium affine strain UTEX FD127	HQ912583	HQ912447	HQ912276
Odontella aurita isolate ECT3619	JX413551	JX413568	JX413585
Odontella mobiliensis isolate ECT3834	KC309499	KC309573	KC309646
Plagiogramma sp. strain HK374	KF701594	KF701603	KF701612
Plagiogramma staurophorum strain ECT3776	HQ912656	HQ912520	HQ912349
Pseudostriatella pacifica strain s0384	AB379680	AB430686	KR048231
Rhabdonema arcuatum strain ECT3898	JN975251	JN975266	JN975280
Rhaphoneis amphiceros strain ECT3828	HQ912673	HQ912537	KC309625
Schizostauron sp. SZCZE421	KT943598	KT943610	KT943622
Stephanopyxis turris strain ECT3828	HQ912657	HQ912521	HQ912350
Striatella unipunctata isolate ECT3874	JX419383	JX419384	JX419385
Synedra ulna strain UTEX FD404	HQ912590	HQ912454	HQ912283
Tabularia cf. tabulata strain CCMP846	HQ912615	HQ912479	HQ912308
Terpsinoe musica strain NHOP43	HQ912682	HQ912546	HQ912370
Toxarium hennedyanum strain ECT3648	HQ912662	HQ912526	HQ912355
Toxarium undulatum strain ECT3802	HQ912654	NC_031425	NC_031425
